# The HtrA chaperone monitors sortase-assembled pilus biogenesis in *Enterococcus faecalis*

**DOI:** 10.1371/journal.pgen.1011071

**Published:** 2024-08-05

**Authors:** Cristina Colomer-Winter, Adeline M. H. Yong, Kelvin K. L. Chong, Mark Veleba, Pei Yi Choo, Iris Hanxing Gao, Artur Matysik, Foo Kiong Ho, Swaine L. Chen, Kimberly A. Kline

**Affiliations:** 1 Department of Microbiology and Molecular Medicine, University of Geneva, Geneva, Switzerland; 2 Singapore Centre for Environmental Life Sciences Engineering, Nanyang Technological University, Singapore, Singapore; 3 School of Biological Sciences, Nanyang Technological University, Singapore, Singapore; 4 Genome Institute of Singapore, Agency for Science, Technology, and Research, Genome #02–01, Singapore, Singapore; The University of Texas Health Science Center at Houston, UNITED STATES OF AMERICA

## Abstract

Sortase-assembled pili contribute to virulence in many Gram-positive bacteria. In *Enterococcus faecalis*, the endocarditis and biofilm-associated pilus (Ebp) is polymerized on the membrane by sortase C (SrtC) and attached to the cell wall by sortase A (SrtA). In the absence of SrtA, polymerized pili remain anchored to the membrane (i.e. off-pathway). Here we show that the high temperature requirement A (HtrA) bifunctional chaperone/protease of *E*. *faecalis* is a quality control system that clears aberrant off-pathway pili from the cell membrane. In the absence of HtrA and SrtA, accumulation of membrane-bound pili leads to cell envelope stress and partially induces the regulon of the ceftriaxone resistance-associated CroRS two-component system, which in turn causes hyper-piliation and cell morphology alterations. Inactivation of *croR* in the OG1RF *ΔsrtAΔhtrA* background partially restores the observed defects of the *ΔsrtAΔhtrA* strain, supporting a role for CroRS in the response to membrane perturbations. Moreover, absence of SrtA and HtrA decreases basal resistance of *E*. *faecalis* against cephalosporins and daptomycin. The link between HtrA, pilus biogenesis and the CroRS two-component system provides new insights into the *E*. *faecalis* response to endogenous membrane perturbations.

## Author summary

To explore the role of the HtrA chaperone/protease in *E*. *faecalis* off-pathway pilus clearance, we deleted *htrA* in an *E*. *faecalis* OG1RF *ΔsrtA* strain known to retain polymerized pili on the cell membrane. Cells in the *ΔsrtAΔhtrA* background are hyper-piliated, possess altered morphology, and are more susceptible to cell envelope-targeting antibiotics as compared to the parent OG1RF strain. RNA sequencing of the *ΔsrtAΔhtrA* strain revealed transcriptional changes reminiscent of a membrane stress response. This response was pilus-dependent and contained several members of the CroR regulon. Inactivation of the response regulator CroR in the *ΔsrtAΔhtrA* background restored (at least partially) piliation and cell morphology but not antibiotic susceptibility, linking CroR for the first time to endogenous cell envelope stress.

## Introduction

Sortase-assembled pili are multi-subunit fibrillar structures that are assembled and covalently attached to the cell wall by sortase enzymes [1]. Conserved among many Gram-positive bacteria, including *Enterococcus faecalis*, *Streptococcus pneumoniae* and *Corynebacterium diphtheriae*, sortase-assembled pili are often important virulence factors that contribute to distinct steps of the infectious process, such as host tissue adherence and biofilm formation. In *E*. *faecalis*, the endocarditis and biofilm-associated pilus (Ebp) operon consists of three pilin genes, *ebpA*, *ebpB* and *ebpC*, and a pilus-specific sortase (*srtC*) whose expression is controlled by a second promoter [2]. Similar to other piliated Gram-positive bacteria, pilin monomers are translocated across the membrane via the Sec secretion machinery, assembled on the membrane into fibers by sortase enzyme C (SrtC), and subsequently attached to the cell wall by the housekeeping sortase enzyme A (SrtA), encoded elsewhere on the chromosome [3]. Pili are typically expressed by only a fraction of the cell population (10–40%) [2,4]. However, the percentage increases in response to host-related environmental stimuli, including serum and bicarbonate [5–8]. Ebp regulation occurs at the transcriptional level, where it is directly, positively regulated by EbpR [9]. Indirectly, *ebp* transcription is activated by the RNase J2 (*rnjB*) and repressed by the quorum-sensing response regulator FsrA, in both cases through regulation of *ebpR* expression [6,9–12].

Though a substantial number of studies have characterized pili and their contribution to infection, most studies focus on pilus regulation and biogenesis under optimal laboratory growth conditions, which may not fully recapitulate physiological conditions within the host. For example, during infection, endogenous cellular stresses or harsh exogenous conditions such as gastric acids, gut bile salts, or oxidative stress give rise to protein folding defects that can compromise the correct assembly and localization of proteins including pili, thus interfering with their function [13]. This was first described in *Escherichia coli*, where misfolded pilins aggregate in the periplasm and are driven ‘off-pathway’ rather than being assembled into pili [14]. Follow-up studies demonstrated that overexpression of misfolded, aggregated pilins activate two different two-component systems (TCS) in *E*. *coli*: Cpx and Bae [14–19]. While both systems synergistically induce rapid expression of the aggregate-resolving Spy chaperone, only Cpx activates DegP, a conserved serine protease of the HtrA (High-Temperature Requirement A) family that degrades misfolded pilins.

It is currently unknown how Gram-positive bacteria respond to and clear aggregated pilins [20]. Analogous to *E*. *coli*, Gram-positive bacteria also encode HtrA proteins, which primarily function as proteases involved in degradation of misfolded proteins during stress conditions [21]. HtrA proteins were also shown to act as chaperones during protein assembly, and during targeting to the cell surface or the extracellular milieu. In this work, we hypothesized that the membrane-anchored serine protease HtrA might also clear off-pathway pili in Gram-positive bacteria. Using *E*. *faecalis* as a model organism for Gram-positive sortase-assembled pilus biogenesis, our studies confirm the role of HtrA in Ebp pili quality control. Accumulation of membrane-bound pili in the absence of HtrA and SrtA perturbed the cell envelope and partially activated the CroRS two-component system, leading to hyper-piliation and changes in cell morphology. Here, we uncover the role of HtrA as a quality control factor of sortase-assembled pili and show how defective pilus sorting to the cell wall interferes with the antibiotic-responding TCS CroRS in a Gram-positive organism.

## Results

### *E*. *faecalis* HtrA is not required for growth, but supports persistence in a wound infection model

**Fig 1:**
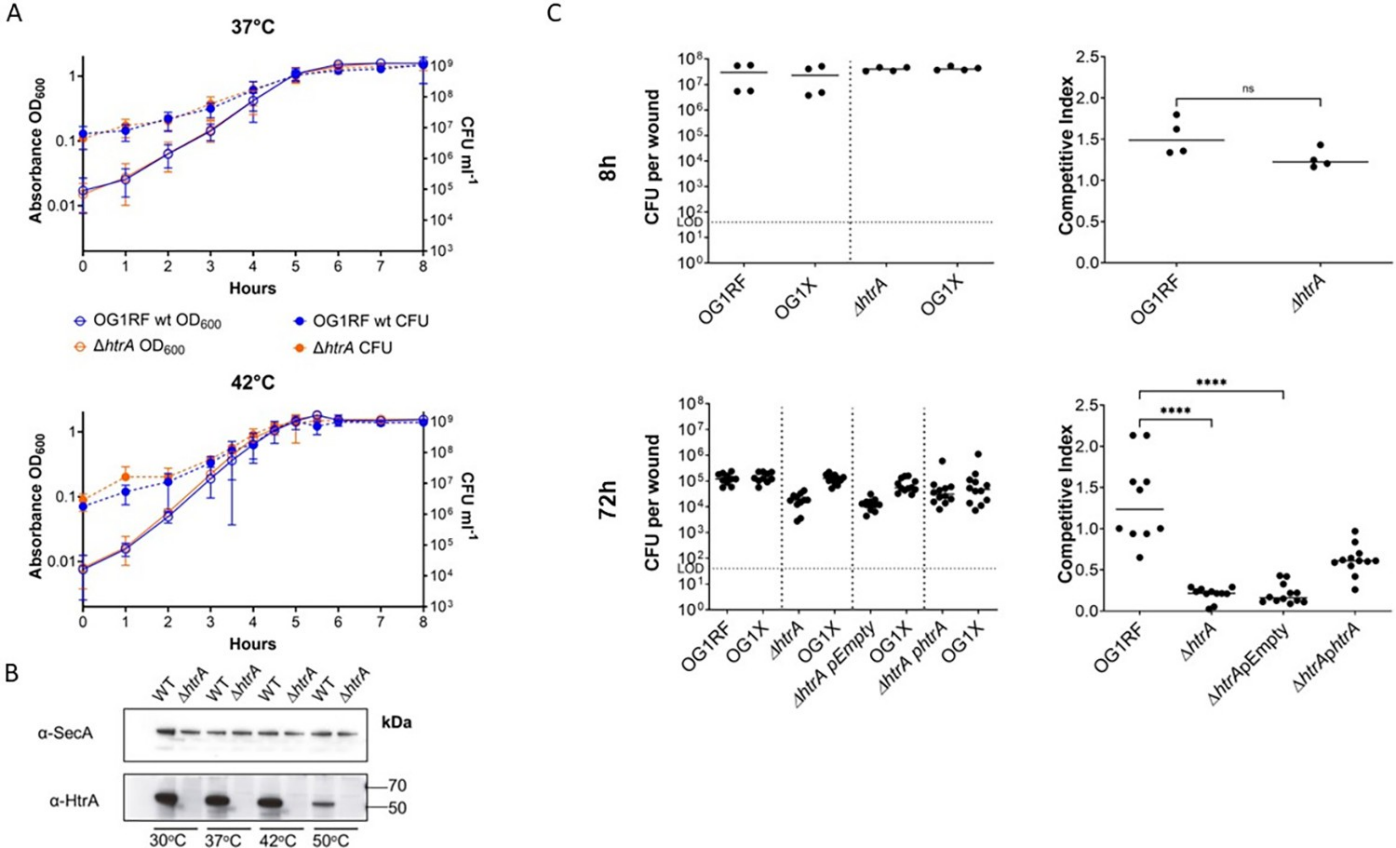
HtrA supports *E*. *faecalis* persistence in wounds. **(A)** Growth phenotypes of *E*. *faecalis* WT and Δ*htrA* strains. Growth curves performed in BHI broth at 37°C (n = 5) and 42°C (n = 2). CFU counts (CFU ml^-1^) are represented as dashed lines; OD_600_ readings are represented as solid lines. Standard deviation is indicated by bars. **(B)** Western blots of whole cell lysates of OG1RF WT and Δ*htrA* grown at different temperatures. To detect HtrA, affinity purified α-HtrA was used. α-SecA was used as a loading control. **(C)** Wounds were infected with a 1:1 ratio of *E*. *faecalis* strains OG1X/OG1RF WT or OG1X/OG1RF Δ*htrA*, at 10^6^ CFU per inoculum, and harvested at 8 hpi or 72 hpi. Recovered bacteria were enumerated on selective media for each strain. Dashed lines separate strain pairs that were co-infected. Each dot represents a mouse. Competitive index was calculated using the final CFU ratio of OG1X with OG1RF WT or Δ*htrA* (output) over the initial CFU ratio of OG1X with OG1RF or Δ*htrA* (input). Solid horizontal line indicates the median. The limit of detection (LOD) of 40 CFU is indicated. For 8h, 4 mice per strain were tested. For 72h, 10–12 mice per strain were used. Statistical analysis was performed using the Kruskal-Wallis test with Dunn’s post-test to correct for multiple comparisons. **** P≤0.0001.

Since the HtrA protein of *E*. *coli* was first discovered as a heat shock-inducible serine protease, and the HtrA homologues of some Gram-positive bacteria were later shown to be heat-inducible such as in *Bacillus subtilis* and *Lactobacillus helveticus* [22–24], we first determined if HtrA is involved in the heat shock response of *E*. *faecalis*. We generated an *E*. *faecalis* OG1RF *ΔhtrA* deletion mutant and screened the strain for growth defects when challenged with heat stress and other stresses known to cause protein misfolding and aggregation [13]. Incubation of WT and *ΔhtrA* cells at 37°C, 42°C, and 50°C for up to 8h did not yield significant differences in planktonic growth or survival among the two strains (**Figs 1A and S1A**). Similarly, no differences between WT and *ΔhtrA* strains were observed regarding 24h and 48h biofilm formation when cells were incubated at 30°C, 37°C or 42°C in BHI or TSBG medium (**S1B Fig**). In parallel, whole lysate immunoblots of cells grown at these temperatures showed that HtrA levels remained steady up to 42°C and decreased at 50°C (**Fig 1B**), altogether suggesting that HtrA is not a major contributor to the heat shock response of *E*. *faecalis* under the tested laboratory growth conditions. Since other types of environmental stresses also cause protein misfolding, we challenged the *E*. *faecalis ΔhtrA* strain with pH, osmotic, and oxidative stress, but no growth differences were observed compared to the parent strain under laboratory growth conditions (**S1C Fig**). To investigate the strain under a more relevant host environment, we tested the *ΔhtrA* mutant for fitness in a competitive mouse wound infection model previously described [25]. Wounds were infected with a total of ~10^6^ CFU consisting of a 1:1 mix of either OG1RF WT/OG1X or *ΔhtrA/*OG1X and harvested after 8h or 72h post-infection. These two time-points were chosen to determine if HtrA might play a role in active replication (8hpi) or in long-term persistence as previously established (72hpi) [25]. We chose OG1X as the comparative strain due to its close genetic kinship to OG1RF (5 different SNPs [26]) and their different antibiotic resistance profile, enabling convenient quantification on selective media [25]. We recovered a median titer of ~10^7^ CFU/mL for all strains after 8h post-infection, indicating no differences in the acute phase of infection (**Fig 1C**). However, after 72h post-infection, we recovered ~1log less *ΔhtrA* (~2.0 x 10^4^ CFU/mL) than OG1RF (~1.3 x 10^5^ CFU/mL). Complementation of *htrA* in *trans* (*ΔhtrA* + p*htrA*) partially restored the competitive index of the *ΔhtrA* strain, suggesting that HtrA might promote *E*. *faecalis* persistence in wounds. Overall, lack of *htrA* appeared to have negligible effects on *E*. *faecalis* growth *in vitro* and played a minor role in *in vivo* wound persistence.

### The HtrA chaperone contributes to removal of off-pathway Ebp pili

**Fig 2:**
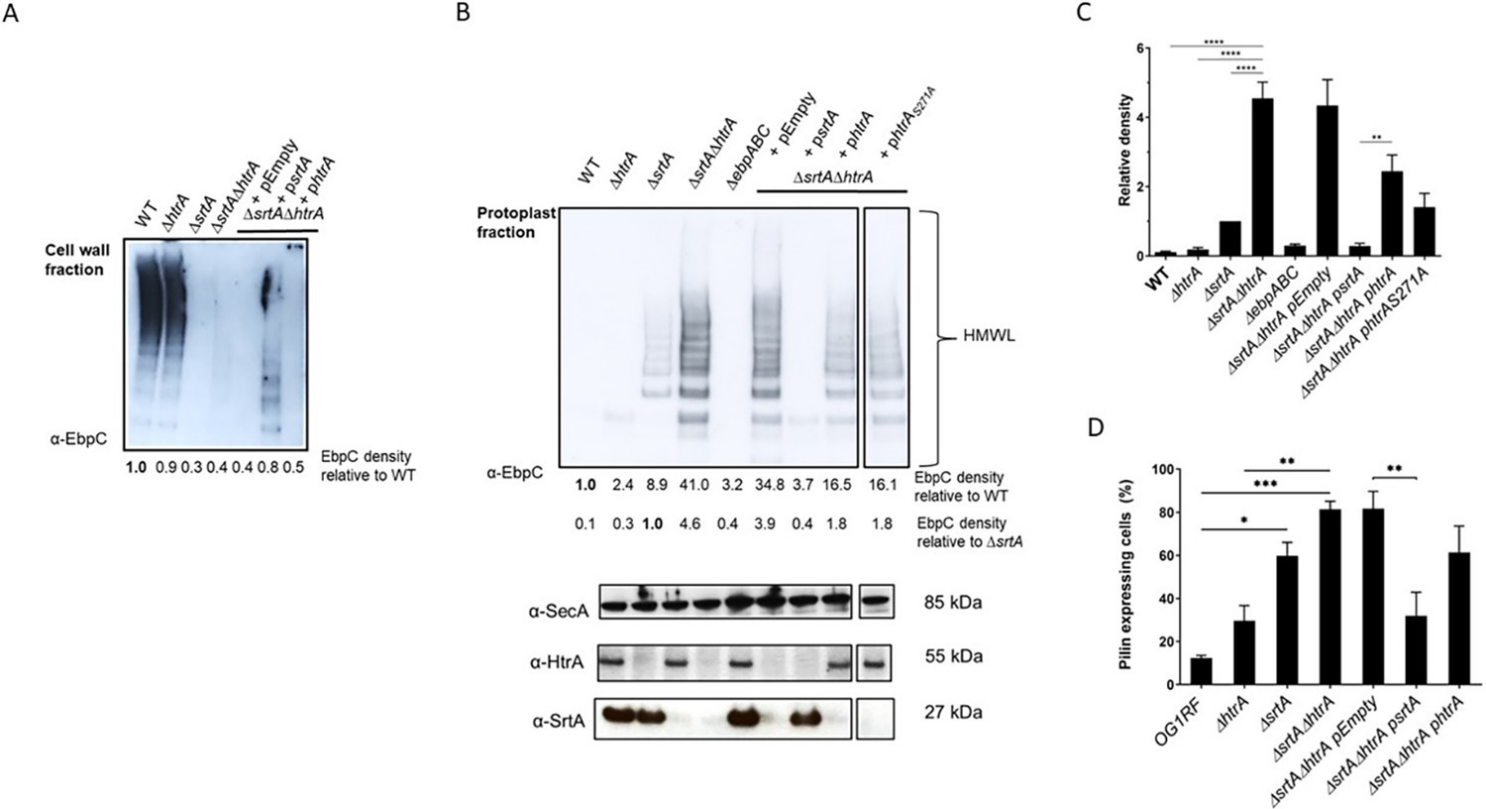
Loss of *htrA* increases EbpC protein levels in the protoplast fraction. **(A)** Immunoblot was performed with α-EbpC on cell wall fractions of WT, *ΔhtrA*, *ΔsrtA*, and *ΔsrtAΔhtrA* strains, as well as *ΔsrtAΔhtrA* carrying p*Empty* (vector control), p*srtA*, or p*htrA*. Blot shows typical pilus high molecular weight ladders (HMWL). Relative EbpC density differences were calculated with WT EbpC expression as the standard. **(B)** Immunoblot was performed with α-EbpC on protoplast fractions of WT, *ΔhtrA*, *ΔsrtA*, *ΔsrtAΔhtrA*, and *ΔebpABC* strains, as well as *ΔsrtAΔhtrA* carrying p*Empty* (vector control), p*srtA*, p*htrA* or p*htrA*_*S271A*_. Top blot shows pilus HMWL and bottom blots show loading and strain controls using α-SecA, α-HtrA and α-SrtA, respectively. Relative EbpC density differences were calculated with WT or *ΔsrtA* EbpC expression as the standard. **(C)** Statistical analysis of relative EbpC density from 4 independent immunoblots, using *ΔsrtA* as the comparison standard, are represented as bar graphs with the standard error of the mean. ** P ≤ 0.01; **** P ≤ 0.0001 **(D)** Statistical analysis of percent pilus-expressing cells of WT, *ΔhtrA*, *ΔsrtA*, *ΔsrtAΔhtrA*, and *ΔsrtAΔhtrA* carrying p*Empty* (vector control), p*srtA* or p*htrA*. Pili were labeled with α-EbpC immune serum and Alexa Fluor 568 secondary antibody. Mean results are represented as bar graphs with standard error of mean. * P < 0.05; ** P ≤ 0.01; *** P≤0.001. Combined data from 5 independent experiments were shown.

In *E*. *faecalis*, the pilus-specific SrtC polymerizes first the pilus tip (EpbA) and the pilus fiber (EbpC), and then links the polymerized structure to the pilus base (EbpB). Following polymerization, pili are transiently retained in the cell membrane via a hydrophobic domain embedded in the EbpB cell wall sorting signal [27,28]. Then, the housekeeping SrtA cleaves the EbpB cell wall sorting signal, generating an acyl-enzyme intermediate, and finally anchors polymerized pili on the cell wall. In *E*. *faecalis*, there are currently two models for SrtA-mediated anchoring of pili to the peptidoglycan layer. In one model, SrtA covalently links pili to a lipid II cell wall precursor at the cell septum [28]. In the second model, pili are alternatively anchored to mature peptidoglycan at the cell hemispheres [29]. In this scenario, pili might migrate within the membrane from the cell septum where polymerization occurs to the cell hemispheres. Previous studies confirmed that in the absence of SrtA (*ΔsrtA*), pili are no longer found in the cell wall fraction. Instead, pili can be found in the protoplast fraction, indicating that polymerized pili remain anchored to the cell membrane [28,29]. Since the main HtrA homolog in *E*. *coli*, DegP, has been shown to remove misfolded pili in *E*. *coli* [14], we asked whether HtrA might be similarly involved in pili biogenesis in enterococci. Specifically, we hypothesized that in *E*. *faecalis*, HtrA might contribute to clearing aberrant pili, decreasing the total number of membrane-bound pili. Thus, we predicted that deletion of *htrA* in the *ΔsrtA* background (*ΔsrtAΔhtrA*) would lead to a more significant accumulation of pili on the cell membrane. To test this, we performed immunoblot analysis on cell wall and protoplast fractions of wild type (WT), *ΔsrtA*, *ΔhtrA*, and *ΔsrtAΔhtrA* strains with anti-EbpC immune serum. As expected for sortase-dependent cell wall-anchored pili [28], the major pilin subunit EbpC was found in the cell wall fraction of the WT strain (**Fig 2A**) and absent in the protoplast fraction (**Fig 2B and 2C**). In line with previous results, EbpC was absent from the cell wall fraction of *srtA* mutants (**Fig 2A**) and remained instead in the protoplast fraction (**Fig 2B and 2C**). Single deletion of *htrA* did not alter EbpC cell fraction localization as the pilus subunit was found in the cell wall fraction similar to the WT strain (**Fig 2A**). Interestingly, we found that EbpC levels were 4.6-fold higher in the protoplast fraction of the *ΔsrtAΔhtrA* mutant strain than in the *ΔsrtA* strain, whereas expression of a control protein (SecA) was unaffected (**Fig 2B and 2C**). We observed similar trends with minor subunits EbpA and EbpB (**S2A Fig**), and complementation of either *srtA* or *htrA* in the *ΔsrtAΔhtrA* background restored, at least in part, pilus levels and cell fraction localization (**Fig 2A and 2B**).

HtrA enzymes often display dual chaperone and protease functions [30,31]. Since Ebp remained bound to the protoplast fraction of the single *ΔsrtA* and the double Δ*srtAΔhtrA* strains, but EbpC levels were significantly higher upon *htrA* inactivation (**Fig 2B and 2C**), we hypothesized that the HtrA serine protease activity may directly degrade membrane-bound Ebp. To test this, we sought to inactivate the protease function without altering the chaperone function. The proteolytic activity of HtrA enzymes is dependent on a highly conserved serine residue found in HtrA homologs of bacteria and mammals alike, including humans [32]. The importance of the serine residue for protease function has been previously confirmed in Gram-positive *S*. *pneumoniae*, *Listeria monocytogenes*, and *Bacillus anthracis* [32–34]. Thus, we designed an HtrA expression plasmid with a single amino-acid change in the conserved serine (S271A) of the proteolytic active site of the enzyme (p*htrA*_S271A_). When assessed using the casein agar plate assay, the Δ*htrA* strain was mildly attenuated in extracellular protease activity when compared to the WT strain (**S2B and S2C Fig**). An OG1RF Δ*gelE* strain lacking one of the main extracellular metalloproteases of *E*. *faecalis* (GelE) was added as a control [35]. While complementation of *htrA* in the Δ*htrA* strain restored extracellular protease activity to WT levels, complementation with the catalytic variant *HtrA*_S271A_ failed to do so, confirming that mutation of the conserved catalytic serine inactivated the protease activity of HtrA (**S2B and S2C Fig**). Immunoblot analysis revealed a decrease in EbpC levels in the Δ*srtA*Δ*htrA* strain complemented with p*htrA*_S271A_, similar to Δ*srtA*Δ*htrA* complemented with the wild-type p*htrA* (**Fig 2B and 2C**), indicating that the HtrA protease activity is dispensable for pili processing and that it’s likely the chaperone activity that is sufficient to remove the accumulated pili from the cell membrane.

Approximately 10–40% of WT cells grown under laboratory conditions in TSBG (10–20%) or BHI (20–40%) media express Ebp pili [2,4]. Though EbpR-dependent regulation of *E*. *faecalis* pili is well characterized, it is currently unknown why only a subpopulation of cells express pili. Inactivation of *srtA* was previously shown to correlate with increased total pilus abundance [28]. However, it was unknown whether the increased Ebp levels observed on the immunoblot of *ΔsrtAΔhtrA* cells reflected the frequency of piliated cells in the total cell population, or hyper-piliation on a single cell level. Immunofluorescence microscopy of EbpC labeled cells grown in TSBG revealed that ~80% of *ΔsrtAΔhtrA* cells in the population expressed pili as compared to WT (~15%), while *ΔsrtA* (~60%) and *ΔhtrA* (~30%) displayed incremental increases in total population piliation (**Fig 2D**). Complemented strains reverted to single mutant piliation levels. To determine if the increase in pili content of the *ΔsrtAΔhtrA* strain is due to hyper-piliation on the single-cell level (i.e. more pili or longer pili on individual cells) or on the population-level (i.e. more cells expressing pili), we performed immunofluorescence staining and quantified the percentage of Ebp^+^ cells within the population as well as the mean fluorescence intensity of single Ebp^+^ cells (**S3 Fig**). No significant differences in fluorescence intensity were observed between individual cells of the WT and the double mutant strain, strongly suggesting that hyper-piliation is mainly occurring at the population level. These results indicate that efficient pilus cell wall-anchoring by SrtA and monitoring by HtrA is an important aspect in the regulation of cell piliation in a given population.

### Accumulation of membrane-bound pili elicit broad transcriptional changes

To investigate the transcriptional response to accumulation of membrane-bound pili, we performed RNA sequencing of OG1RF WT, Δ*srtA*, Δ*htrA* and Δ*srtA*Δ*htrA*. When compared to OG1RF WT, single inactivation of *htrA* led to differential expression of 4 genes (3 hypothetical genes and flavocytochrome C), while deletion of *srtA* altered transcription of 11 genes, including repression of PTS sugar transport genes, and up-regulation of arginine metabolism and the quorum sensing transcriptional regulator *fsrA* (**S4 Table**). Strikingly, simultaneous deletion of *srtA* and *htrA* changed the expression of 305 genes (107 up-regulated, 198 down-regulated).

**Fig 3:**
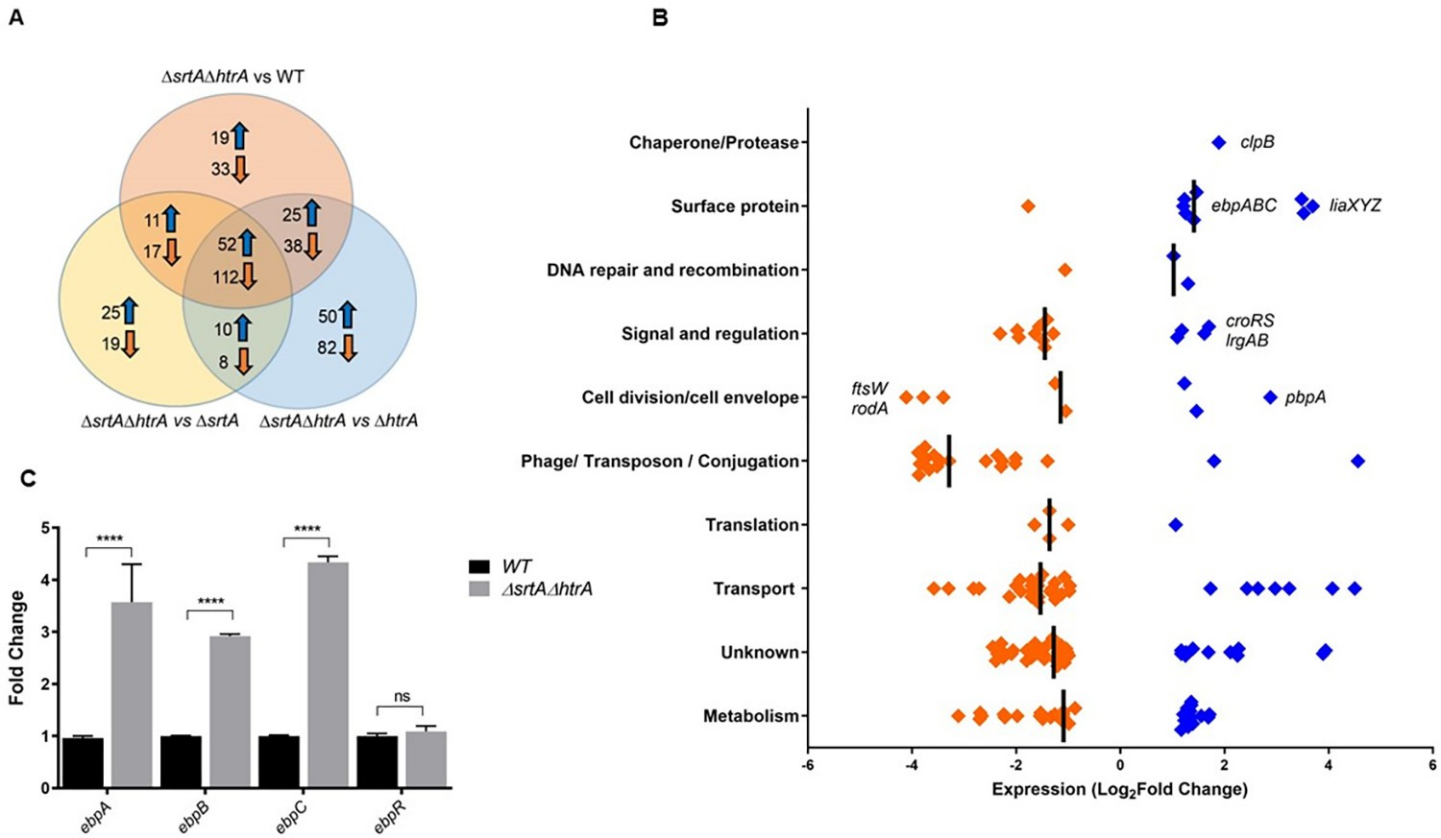
**Global transcriptional changes due to absence of *htrA* and *srtA* (A)** Venn diagram showing a total of 164 genes found to be differentially regulated specifically during pili accumulation on the cell membrane (i.e. in the absence of both *srtA* and *htrA*). Genes that are only found in one or two of the indicated groups are in the non-overlapping regions. The total number of differentially expressed genes in each comparison is indicated inside each group. **(B)** The log_2_ fold change in mRNA levels of the core 164 genes that are differentially expressed in the *ΔsrtAΔhtrA* strain. Values displayed as a dot plot correspond to differences between *ΔsrtAΔhtrA* and *ΔsrtA*. Significant genes were determined by the Bioconductor package EdgeR (P < 0.05; FDR < 0.05). Genes with negative log_2_ fold change are colored orange; genes with positive log_2_ fold change are colored blue. Genes of interest were labeled for easier identification. **(C)** qRT-PCR analysis of *ebpABCR* expression in the WT (black bars) and *ΔsrtAΔhtrA* (grey bars). qRT-PCR was performed in biological triplicates and analyzed by the ΔΔ_CT_ method, using *gyrB* as a housekeeping gene. Fold change indicates the change in *ebpA*, *ebpB*, *ebpC* and *ebpR* transcription compared to WT. Statistical analysis was performed by 2-way ANOVA and Tukey’s multiple comparison tests using GraphPad. **** P ≤ 0.0001, P ≥ 0.05 differences not significant (ns).

Accumulation of misfolded, aggregated pili has been shown to perturb the inner cell membrane of *E*. *coli* [14–16]. In *E*. *coli*, inner membrane perturbations are sensed by the Cpx two-component system (TCS), which orchestrates a stress response to return to membrane homeostasis. The Cpx regulon includes repression of non-essential lipoproteins (such as solute transport systems), and concomitant activation of protein folding enzymes (such as DegP and Spy) and peptidoglycan-modifying enzymes [15,36]. We hypothesized that accumulation of membrane-bound pili could similarly perturb the cell membrane of *E*. *faecalis*. We thus searched for commonalities between the *E*. *coli* Cpx regulon and the *E*. *faecalis* transcriptional response to aggregated, accumulated pilins. To streamline our efforts, pairwise transcriptome comparisons between the double mutant strain and either the WT or the single *htrA* or *srtA* mutant strains identified 164 genes that were differentially regulated in all three comparisons (p<0.05; FDR<0.05), suggesting that these genes may be specifically and consistently involved in the response to accumulation of membrane-bound pili (**Fig 3A and S5 Table**). We classified these 164 consensus genes into predicted functional groups for further analysis. The log_2_ fold change in mRNA levels in *ΔsrtAΔhtrA* are displayed relative to *ΔsrtA* as an example and are shown as a dot plot (**Fig 3B**).

In line with the *E*. *coli* Cpx inner membrane stress response [15,37,38], a significant number of repressed genes clustered in the transport and metabolism categories, including non-essential sugar and amino acid transporters. Moreover, genes involved in peptidoglycan turnover were induced, including the penicillin-binding protein *pbpA* (~1.7 log_2_-fold), the murein hydrolase regulators *lrgAB*, and the *croRS* TCS (~ 1.5 log_2_-fold). CroRS [short for “ceftriaxone resistance”] typically regulates penicillin-binding proteins in response to antibiotic-induced cell wall damage [39–41]. In the protease/chaperone category we only observed upregulation of *clpB* (~1.7 log_2_-fold), a key chaperone that removes protein aggregates in several bacterial pathogens [13,42]. In contrast to *E*. *coli*, differentially expressed genes that clustered in the translation category were generally repressed. Overall, these results suggested that off-pathway pili also cause cell envelope perturbations in *E*. *faecalis*, but that the coping strategies utilized by this Gram-positive bacterium partially differ from *E*. *coli*. Transcriptional analysis of the double *ΔsrtAΔhtrA* strain harbored additional findings supporting membrane stress. For example, accumulation of off-pathway pili led to strong upregulation of the *liaXYZ* operon (~3 log_2_-fold) involved in modulation of the membrane stress-sensing TCS LiaFSR. Differential expression of genes involved in peptidoglycan synthesis such as putative *ftsW* (OG1RF_11070) and *rodA* (OG1RF_11071) was also detected. Consistent with the increased pilus expression observed in this strain, transcription of the pilin genes *ebpA*, *ebpB* and *ebpC* was significantly induced (~1.6 log_2_-fold RNA-Seq, ~3.6-fold qRT-PCR) in the *ΔsrtAΔhtrA* mutant (**Fig 3B and 3C**). Transcript levels of the positive regulator *ebpR*, as well as of *ebpR*-regulating *rnjB* and *fsrA*, were unchanged in the *ΔsrtAΔhtrA* strain (**S4 Table**), suggesting that induction of the *ebp* locus may be regulated independently of EbpR. Finally, we compared the transcriptomes of Δ*srtA*Δ*htrA* and Δ*srtA*Δ*ebpABC*Δ*htrA* (**S6 Table**) focusing on the core gene list identified above (S5 Table). Deletion of pilus genes (*ebpABC*) restored or partially returned transcription closer to WT levels of ~70 genes, including the murein hydrolase regulators *lrgAB*, the *croS* histidine kinase, the *liaXYZ* accessory operon of the membrane stress-sensing TCS LiaFSR, and a significant amount of solute transporters (**S7 Table**). Other genes remained dysregulated in the Δ*srtA*Δ*ebpABC*Δ*htrA* strain, indicating that deletion of *htrA* and *srtA* has other effects beyond pili accumulation.

### Accumulation of off-pathway pili partially mobilizes the CroR regulon

**Fig 4:**
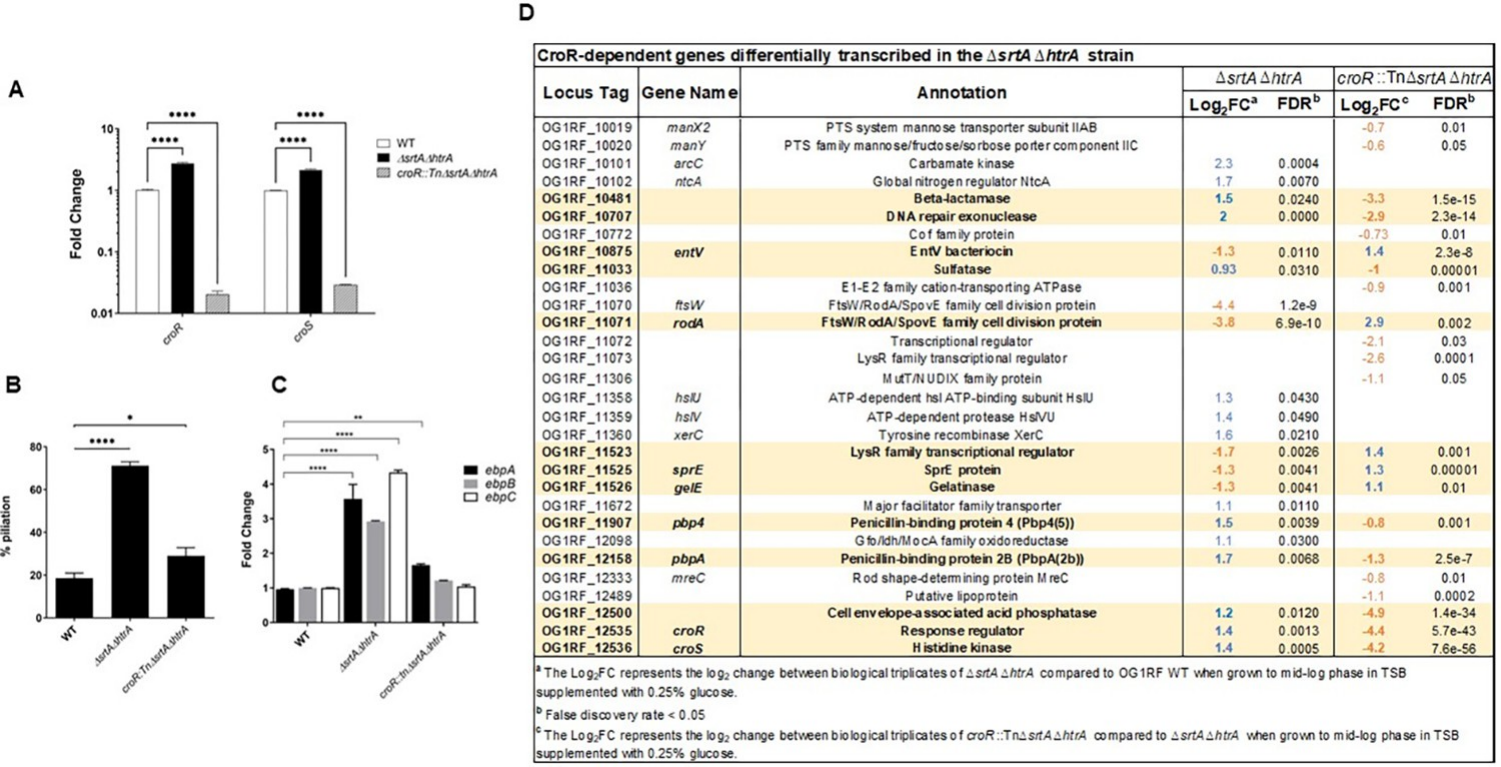
Accumulation of off-pathway pili on the cell membrane activate the CroRS system. (**A**) qRT-PCR analysis of *croRS* expression in the WT (white bars), *ΔsrtAΔhtrA* (black bars), and *croR*::*tnΔsrtAΔhtrA* (light grey bars). qRT-PCR was performed in biological triplicates and analyzed by the ΔΔ_CT_ method, using *gyrB* as a housekeeping gene. Fold change indicates the change in *croR* and *croS* transcription compared to WT. c*roR*::*tnΔsrtAΔhtrA* was used as the negative control for *croRS* transcription. Statistical analysis was performed by the 1-way ANOVA and Tukey’s comparison test using GraphPad. **** P ≤ 0.0001. (**B**) Statistical analysis of pili expressing cells labeled with α-EbpC immune serum and Alexa Fluor 568 secondary antibody. Results are represented as bar graphs with standard error of mean. Combined data from three independent experiments were shown. Statistical analysis was performed by the 2-way ANOVA and Tukey multiple comparison test using GraphPad. * P ≤ 0.05; *** P ≤ 0.001; **** P ≤ 0.0001; P > 0.05, differences not significant (ns). (**C**) qRT-PCR analysis of *ebpA* (black bars), *ebpB* (light gray bars) and *ebpC* (white bars) expression in the WT, *ΔsrtAΔhtrA* and *croR*::*tnΔsrtAΔhtrA* strains. qRT-PCR was performed in biological triplicates and analyzed by the ΔΔ_CT_ method using *gyrB* as a housekeeping gene. Fold change indicates the change in *ebpABC* gene transcription compared to WT. Statistical analysis was performed by the 2-way ANOVA and Tukey multiple comparisons test using GraphPad. **** P ≤ 0.0001. (**D**) List of CroR-dependent genes identified by Timmler *et al* in bacitracin-treated OG1 [39] that are differentially expressed in the *ΔsrtAΔhtrA* strain (compared to OG1RF WT), in the *croR*::*tnΔsrtAΔhtrA* strain (compared to *ΔsrtAΔhtrA)*, or in both. Log_2_FC values are indicated in blue for upregulated genes and orange for downregulated genes. Genes highlighted in bold font and yellow background are restored, at least in part, to WT levels upon *croR* inactivation.

Since TCS such as Cpx and Bae sense pilus aggregates in *E*. *coli* [14,16–19], and transcription of the *croRS* TCS was induced (~1.4 log_2_-fold RNA-Seq, ~2.5-fold qRT-PCR) in the *E*. *faecalis ΔsrtAΔhtrA* strain (**Figs 3B and 4A**), we sought to determine if CroRS regulated at least part of the pilus-responsive response. We constructed a *croR*::*tnΔsrtAΔhtrA* triple mutant strain and performed immunofluorescence pili staining as part of the initial characterization. Inactivation of *croR* significantly decreased piliation levels in the cell population (*croR*::*tnΔsrtAΔhtrA* (~36%)) as compared to *ΔsrtAΔhtrA* (60–80%) (**Figs 4B and S4B**). In line with pilus surface expression, inactivation of *croR* also decreased *ebp* transcription levels as quantified per qRT-PCR (**Fig 4C**). Complementation of *croR*::*tnΔsrtAΔhtrA* with *croR* failed to revert the observed phenotypes; however, complementation of *croRS* on an expression plasmid (p*croRS-2xHA*) caused again hyper-piliation (**S4B Fig**).

Basal transcription of TCS genes, including Cpx and CroRS, under unstressed conditions is typically low to avoid nonspecific activation of their regulon [41,43,44]. When activated (i.e. usually phosphorylated) by a given signal, TCS response regulators can activate their own transcription in a positive feedback loop until the stress is resolved [43]. Upregulation of *croRS* in the *ΔsrtAΔhtrA* strain suggests that membrane overloading with off-pathway pili might activate the CroRS system. If true, we would expect to see at least part of the CroR regulon embedded in the transcriptome of the *ΔsrtAΔhtrA* mutant, and inactivation of CroR would restore CroR-dependent genes to WT levels. We performed RNA-Seq of the *croR*::*tnΔsrtAΔhtrA* triple strain and compared it to the transcriptome of the *ΔsrtAΔhtrA* double mutant (**S8 and S4 Tables**), while also scanning for the signature of the CroR regulon in the *ΔsrtAΔhtrA* strain. Of note, to avoid strain specific differences we used as our reference the CroR regulon identified using bacitracin-treated OG1 (the ancestral strain of OG1RF) [39,45]. Our analysis uncovered that ~24% of CroR-dependent genes (21 out of 88 genes) were differentially expressed in the *ΔsrtAΔhtrA* strain (**Fig 4D**). Of these, ~52% were restored, at least in part, to wild-type levels upon *croR* inactivation, including hallmark CroR-dependent genes such as the cephalosporin low affinity penicillin-binding proteins *pbp4* and *pbpA* [39].

### Aberrant cell morphology of the *ΔsrtAΔhtrA* strain is Ebp- and CroR-dependent

**Fig 5:**
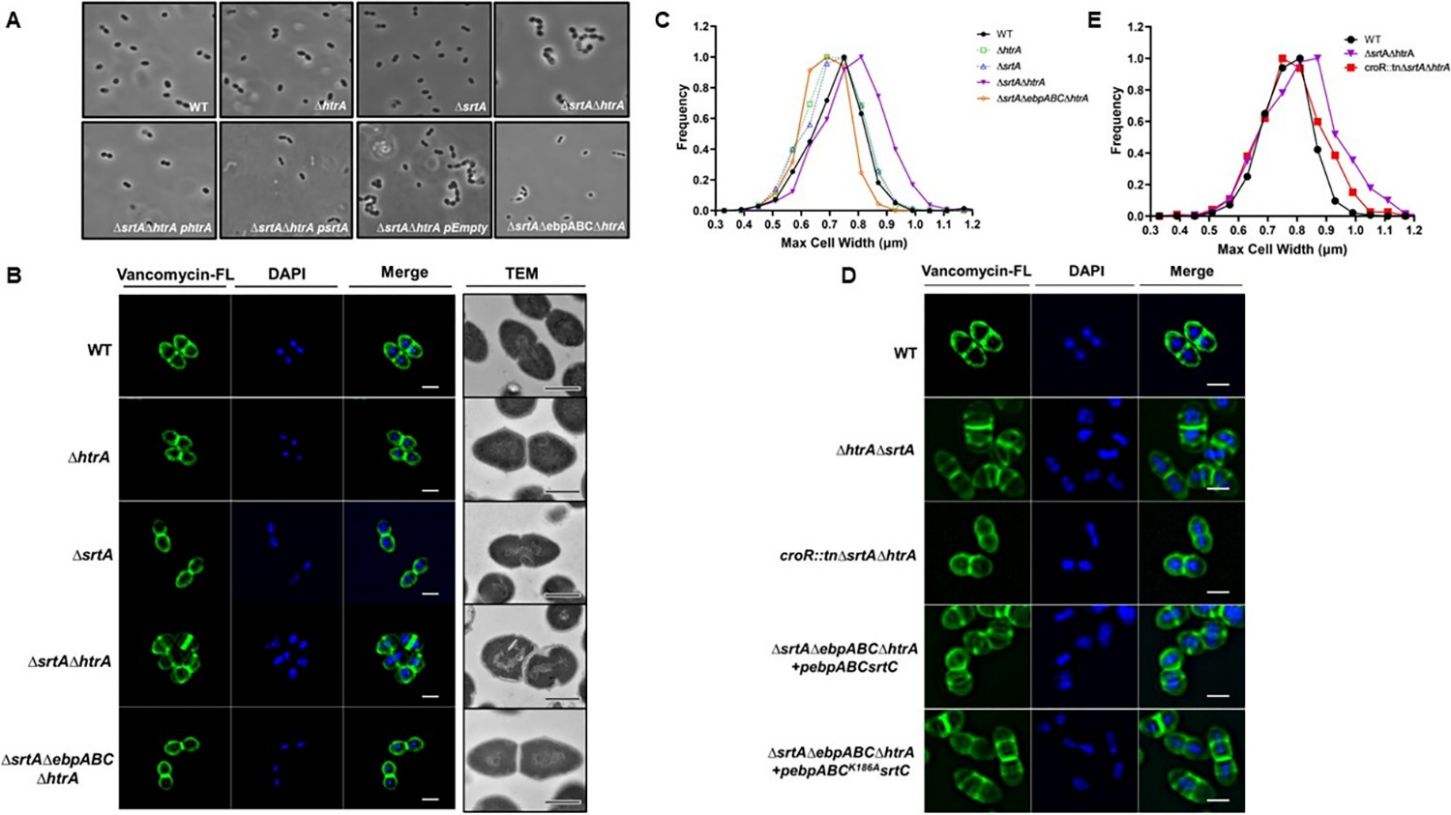
Aberrant cell morphology of the *ΔsrtAΔhtrA* mutant is pilus- and CroR-dependent. (**A**) The morphology of the indicated *E*. *faecalis* strains was visualized at a magnification of 100X with a phase contrast microscope. Representative images are shown. (**B, D**) The cell wall of log-phase growing cells was stained with Vancomycin-FL conjugate (green). DAPI (blue) was used to visualize DNA. The same representative images are shown in panels B and D for the WT strain, for ease of comparison. Bacterial ultrastructures were observed under TEM. Scale bars for immunofluorescence microscopy and transmission electron microscopy represent 1 μm and 500 nm, respectively. (**C, E**) The mid-cell width for each strain was determined and quantified using the MicrobeJ plugin in ImageJ and the distribution of the population is represented by a Gaussian distribution. The data was plotted using Microsoft Excel. Cells that were not in-phase were excluded from the analysis. A total of at least 200 cells was analyzed per strain.

Transcriptomic analysis revealed several putative cell division genes that were differentially expressed in the *ΔsrtAΔhtrA* strain (**Fig 3B**) and that were partially restored in the triple *croR*::*tnΔsrtAΔhtrA* strain (**Fig 4D**). To determine the presence of cell morphology defects, we first performed phase contrast microscopy of the OG1RF parental strain and the panel of single *htrA*, *srtA* and double mutant strains. A distinct phenotype was observed in a subset of the *ΔsrtAΔhtrA* population, characterized by the presence of chains containing 4–8 joined cells (**Figs 5A and S5A**). The chaining phenotype of *ΔsrtAΔhtrA* could be complemented with p*htrA* or p*srtA*, resulting in reversion to a diplo-ovococcal shape similar to WT (**Fig 5A**). To gain more insight into the morphology defect, which was suggestive of a defect in cell division or cell elongation at the peptidoglycan (PG) level, we visualized PG using the BODIPY FL vancomycin (Van-FL) dye. Of note, vancomycin binds to the terminal D-Ala-D-Ala found on uncrosslinked PG precursors of the entire cell wall, allowing for visualization of peripheral cell wall as well as the septum [46]. In addition to chaining, individual *ΔsrtAΔhtrA* cells appeared shorter and wider than WT (**Fig 5B**), suggesting a defect in cell elongation. In addition, Van-FL labeling revealed aberrant placement of septa across 30% of the *ΔsrtAΔhtrA* cell population (**Figs 5B and S5B**), indicating that cell division was also affected. Further transmission electron microscopy confirmed that some *ΔsrtAΔhtrA* cells had severely deformed septa (**Figs 5B and S5C**). Moreover, these cells lost the ovococcal WT shape and exhibited a more spherical morphology. To quantify the cell shape, we measured the cell width of each strain and plotted it as a distribution curve. The majority of *ΔsrtAΔhtrA* cells had a cell width of ~0.8 μm, while WT, *ΔsrtA* or *ΔhtrA* cells neared 0.7 μm (**Fig 5C**). Altogether, it appeared that the simultaneous absence of *srtA* and *htrA* hampered the ability of the cell to elongate and divide, reminiscent of the LiaFSR-dependent cell membrane stress response in enterococci [47,48].

Considering that *croR* inactivation partially restored transcription of the dysregulated cell division genes of the *ΔsrtAΔhtrA* strain (**Fig 4D**), we hypothesized that inactivation of *croR* in the triple *croR*::*tnΔsrtAΔhtrA* strain would restore the division defects of the *ΔsrtAΔhtrA*. By measuring cell width and performing cell wall staining, we observed that *croR*::*tnΔsrtAΔhtrA* indeed no longer exhibited morphology defects (**Fig 5D and 5E**). Since membrane overloading with pili perturbs the cell envelope and likely triggers activation of the CroR regulon, we deleted the entire *ebp* locus in the *ΔsrtAΔhtrA* background to remove the triggering stress source (quintuple *ΔsrtAΔebpABCΔhtrA* mutant). Identical to *croR* inactivation, deletion of *ebpABC* restored cell morphology to that of the WT strain (**Fig 5A and 5C**). Complementation of *ebpABC* in the quintuple strain triggered once again morphology defects (**Fig 5D**).

While the exact signal sensed by CroRS remains elusive, it has been suggested to be related to cell wall damage [39,40,49]. We have recently shown that in the *ΔsrtAΔhtrA* strain, membrane-bound pili protrude from the cell membrane through the cell wall instead of being anchored to the cell wall [29]. Here, we hypothesized that protrusion of long membrane-anchored pili could potentially perturb the cell wall by physically altering the peptidoglycan layer, activating the CroRS system. To begin to interrogate if CroRS activation was due to membrane protein overloading or peptidoglycan perturbation, we investigated whether accumulation of monomeric pilin subunits that cannot be polymerized into full length pili but remain anchored to the membrane equally induce CroR-dependent morphological defects. This would begin to interrogate if the trigger relies on alterations to the membrane as opposed to alterations due to physical disturbance of the peptidoglycan layer. We used a plasmid with a single amino-acid change in the EbpC pilin-like motif (K186A) that is necessary for pilus fiber polymerization, resulting in the expression of EbpC monomers [28]. Complementation of *ΔsrtAΔebpABCΔhtrA* with genes encoding either monomeric pilin subunits (p*ebpABC*_*K186A*_*srtC*) or polymerized pili (p*ebpABCsrtC*) both gave rise to morphology defects (**Figs 5D and S5D**), indicating that membrane perturbation was likely responsible for CroR activation.

### CroR partially induces cell morphology defects through regulation of RodA

**Fig 6:**
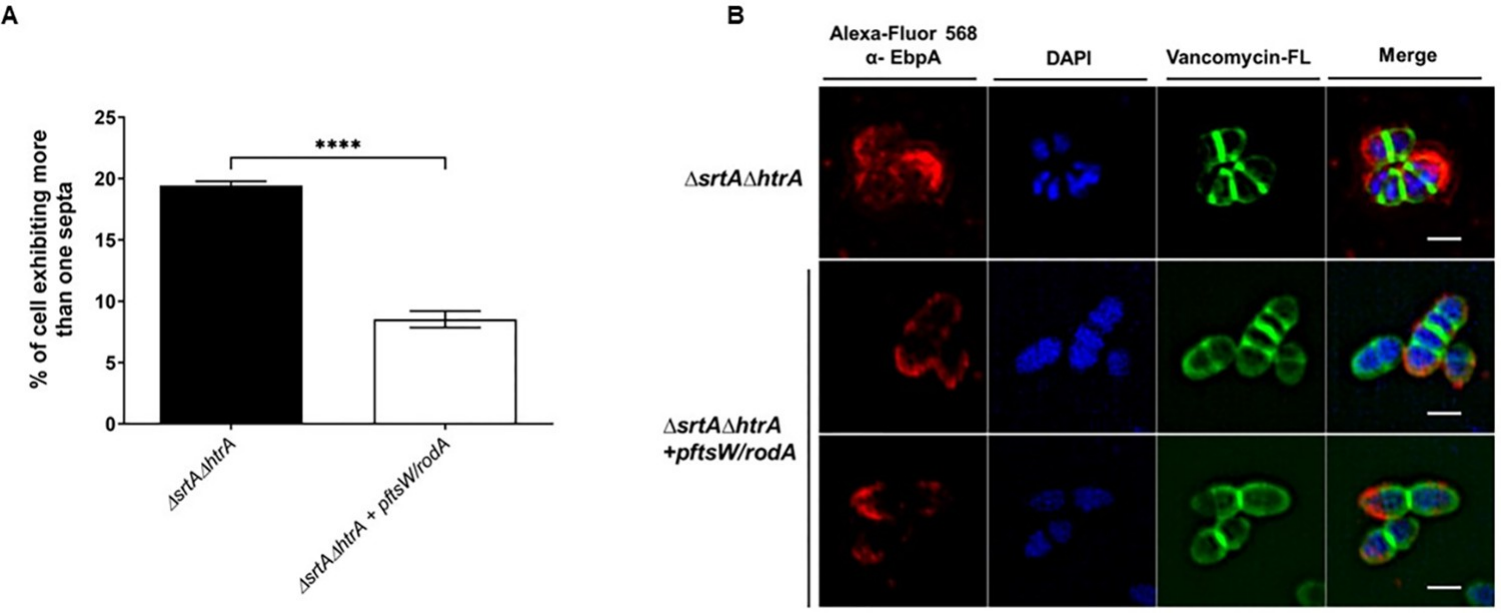
CroR partially induces cell morphology defects through transcriptional regulation of the glycosyltransferase *rodA*. (A) The percentage of cells exhibiting more than 1 septum per cell are represented as bars. The data was plotted using GraphPad Prism. A total of 500 cells were analyzed per strain. Statistical analysis was performed by the unpaired parametric T-test using GraphPad. **** P ≤ 0.0001. (**B**) The cell wall of Δ*srtA*Δ*htrA* and Δ*srtA*Δ*htrA* + p*ftsW/rodA* was stained with Van-FL conjugate (green), blocked and incubated with α-EbpC immune serum coupled to Alexa Fluor 568 antibody (red). DAPI (blue) was used to visualize DNA. Representative images were shown to illustrate the partial morphology restoration. Scale bars represent 1 μm.

The *ΔsrtAΔhtrA* mutant differentially expressed a small group of genes that clustered in the cell division and cell envelope category, including *ftsH*, OG1RF_11070, OG1RF_11071, *pbp4*, *pbpA*, and *pbp2A* (**S4 Table**). Transcription of *pbp4*, *pbpA*, and OG1RF_11071 were restored upon CroR inactivation (**Fig 4D**). We hypothesized that CroR might alter cell morphology defects via control of at least one of these genes. While Pbp4 and PbpA are penicillin-binding proteins linked to cell wall homeostasis, the function of OG1RF_11071 was previously unknown. OG1RF_11071 and its neighboring gene OG1RF_11070 were both annotated as FtsW/RodA/SpovE family cell division proteins. Based on BLASTp searches, the product of OG1RF_11071 shared ~41% identity with the protein sequence of *rodA* in *Streptococcus oralis*, and OG1RF_11070 shared ~36% identity with *ftsW* in *Streptococcus agalactiae*. We will refer to them as *ftsW* and *rodA* from here on for brevity. FtsW is a universally conserved peptidoglycan polymerase essential for septal cell wall assembly [50], while RodA is a highly conserved glycosyltransferase involved in cell wall morphogenesis [51]. Both enzymes interact with penicillin-binding proteins to control cell shape and septation in other bacteria [50–53], and are predicted to be co-transcribed in a single operon in *E*. *faecalis*. We designed an expression plasmid containing both genes (p*ftsW/rodA*) and expressed it in *trans* in the *ΔsrtAΔhtrA* strain. The morphological defect (as measured by multiple septa) was partially alleviated in *ΔsrtAΔhtrA* p*ftsW/rodA*, with 9.5 ± 3% of cells still exhibiting the morphology defect, as compared to 19.3 ± 0.7% in the double *ΔsrtAΔhtrA* strain (**Fig 6A and 6B**). Since *rodA* but not *ftsW* transcription was altered in the triple *croR*::*tnΔsrtAΔhtrA* strain, these results suggest that CroR partially induces morphology changes through regulation of RodA. However, the morphology defect is likely multi-factorial and might involve control of FtsW and partner penicillin-binding proteins such as Pbp4 and PbpA [53].

### Presence of the non-cognate CisS histidine kinase in the absence of CroS sustains cell morphology defects

**Fig 7:**
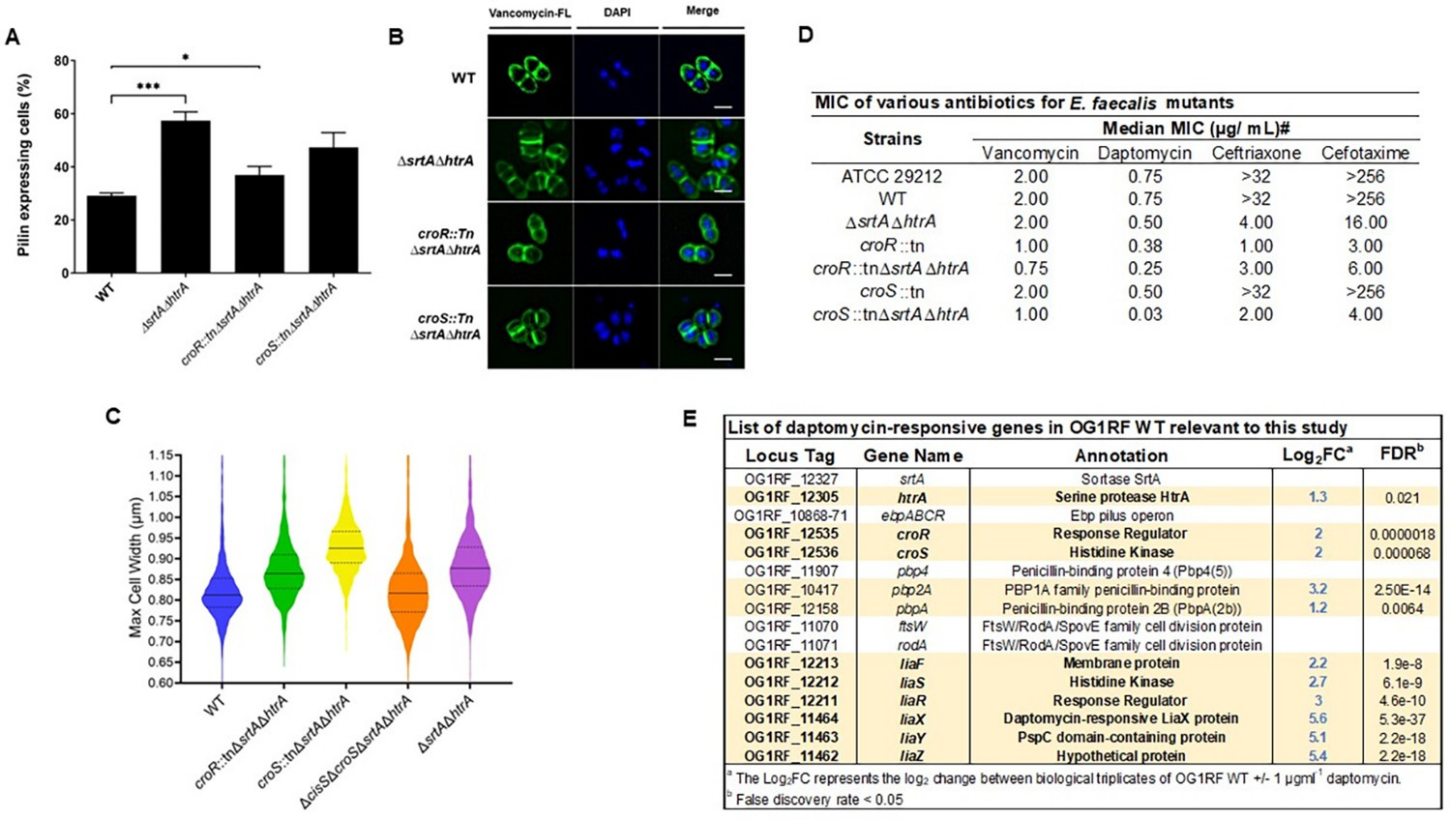
CisS supports cell morphology defects and lack of *srtA* and *htrA* leads to antibiotic susceptibility. **(A)** Statistical analysis of pili expressing cells labeled with α-EbpC immune serum and Alexa Fluor 568 secondary antibody. Results are represented as bar graphs with standard error of mean. Combined data from three independent experiments were shown. Statistical analysis was performed by the 2-way ANOVA and Tukey multiple comparison test using GraphPad. *** P ≤ 0.001; **** P ≤ 0.0001; P > 0.05, differences not significant (ns). **(B)** The cell wall was stained with Vancomycin-FL conjugate (green). DAPI (blue*)* was used to visualize DNA. Representative images were shown. Scale bars represent 1 μm **(C)** The maximum cell width of the various mutant strains was measured and quantified using MicrobeJ plugin in ImageJ and are represented as violin plot. Cells that were not in-phase were excluded from the analyses. A total of at least 900 cells were sampled per strain. Dashed line represents quartiles; horizontal solid line represents the median **(D)** Minimal inhibitory concentration of various antibiotics for *E*. *faecalis* mutants. ^#^ indicates the median MICs reported from 2 biological replicates. ATCC 29212 is the positive control strain that fits the standard MIC of gentamicin (4–16 μg/mL) **(E)** List of selected genes relevant to this study and their log_2_-fold change in transcription in OG1RF WT treated with subinhibitory concentrations of daptomycin. Differentially expressed genes are highlighted in bold font and yellow background.

Since CroR can be phosphorylated by the non-cognate histidine kinase CisS in the absence of CroS [49], we asked if CroR-CisS crosstalk was also present in OG1RF during accumulation of membrane-bound pili. First, we constructed a triple *croS*::*tnΔsrtAΔhtrA* strain and examined cells for restoration of piliation and morphology defects as a readout for CroR activation. Inactivation of the cognate *croS* histidine kinase only partially restored piliation levels and did not revert morphology defects (**Fig 7A–7C**), suggesting that CroR may still be activated by CisS, hence resulting in morphological defects. To further address this hypothesis, we created a quadruple *ΔcisSΔcroSΔsrtAΔhtrA* deletion mutant to remove both sensor histine kinases and quantified the cell width of the panel of strains. The *ΔsrtAΔhtrA* and *croS*::*tnΔsrtAΔhtrA* strains were on average wider (~0.90 μm and ~0.93 μm) than the WT and *croR*::*tnΔsrtAΔhtrA* strains (~0.84 μm and ~0.86 μm). Double inactivation of the two histidine kinases in the *ΔcisSΔcroSΔsrtAΔhtrA* strain restored average cell width to ~0.83 μm (**Fig 7C**), suggesting the existence of CroR-CisS crosstalk under these conditions.

### Absence of *srtA* and *htrA* leads to cephalosporin and daptomycin antibiotic susceptibility

*E*. *faecalis* is a leading cause of antibiotic-resistant infections, partially due to an intrinsic resistance to cell wall-active antibiotics [54]. One of the systems used by *E*. *faecalis* to sustain high basal resistance to β-lactams is the CroRS TCS, in which phosphorylated CroR upregulates cephalosporin tolerant penicillin-binding proteins such as Pbp4 and PbpA in response to antibiotic treatment [39,40,49,55]. Since aberrant accumulation of Ebp pili triggered activation of CroRS in the *ΔsrtAΔhtrA* strain, we hypothesized that the *ΔsrtAΔhtrA* strain would be more resistant to cell wall-active antibiotics such as cephalosporins. Thus, we determined the minimum inhibitory concentration (MIC) of antibiotics whose resistance is dependent upon CroR activation (e.g. vancomycin, ceftriaxone, and cefotaxime) in the *croR*, *croS* and *ΔsrtAΔhtrA* mutant strains (**Fig 7D**) [41,49,56]. In line with previous studies, the *croR*::*tn* strain was hyper-susceptible to ceftriaxone and cefotaxime, while the *croS*::*tn* strain was not [49]. Surprisingly, the *ΔsrtAΔhtrA* strain was highly sensitive to these cephalosporins (*ΔsrtAΔhtrA* 4 μg/mL and 16 μg/mL, WT>32 μg/mL and >256 μg/mL) and inactivation of either *croR* or *croS* in this background further decreased antibiotic MIC comparable to the single *croR*::*tn* strain. Vancomycin susceptibility was mainly observed in the context of *croR* inactivation.

Our results suggested that CroRS responds to endogenous, pilus-dependent membrane perturbation. We hypothesized that CroRS could also respond to exogenous membrane perturbations. The last resort antibiotic daptomycin perturbs the cell membrane, representing an exogenous source of membrane stress [57]. RNA-Seq transcriptome analysis performed in our lab of WT OG1RF treated with subinhibitory concentrations of daptomycin induced expression of *htrA* and *croRS* (**Fig 7E and S9 Table**), suggesting that CroRS might indeed also respond to exogenous membrane stress. Thus, to determine if CroRS contributes to daptomycin resistance, we determined the daptomycin MIC of the panel of mutant strains. The single *croR*::*tn* and *croS*::*tn*, as well as the double *ΔsrtAΔhtrA* strain were mildly more daptomycin sensitive. Strikingly, the triple *croS*::*tnΔsrtAΔhtrA* strain displayed a daptomycin hyper-susceptibility (*croS*::*tnΔsrtAΔhtrA* 0.032 μg/mL, WT 0.75 μg/mL) that was not apparent in the triple *croR*::*tnΔsrtAΔhtrA* (0.25 μg/mL). Overall, our results indicate that dual inactivation of *srtA* and *htrA* reduces the cephalosporin and daptomycin MIC of *E*. *faecalis*, and that inactivation of the *croS* histidine kinase gene in this background further increases daptomycin susceptibility.

## Discussion

During colonization and infection, bacteria face unfavorable environmental conditions such as acidic pH and bile salts that cause proteins to misfold and aggregate, leading to substantial proteotoxic stress if left unresolved [13]. This is especially true for surface exposed proteins that directly interact with the host milieu. To avoid accumulation of defective proteins that might impair crucial cellular processes, bacteria evolved protein quality control systems that clear aberrant proteins from the cell surface [21]. To better understand how enterococci adapt to proteotoxic cell envelope stress, we used the *E*. *faecalis* Ebp pilus system. Here, we show that the HtrA chaperone acts as a protein quality control factor involved in clearance of aberrant membrane-anchored pili. By ensuring that pili do not accumulate on the membrane, HtrA acts as a first line of defense that prevents perturbation of membrane homeostasis and unproductive activation of CroRS, which promotes continued pili expression (i.e. increased proteotoxic stress) and profound cell morphology alterations.

The HtrA protease family plays a key role in protein quality control, including degradation of misfolded and mislocalized pili [21]. Here we propose that in *E*. *faecalis*, HtrA acts as a precautionary measure against mislocalization of pili by monitoring pilus biogenesis and SrtA-dependent sorting, thus intervening only in the presence of protein processing defects. This is exemplified by the fact that absence of *htrA* alone does not affect pilus biogenesis under optimal laboratory growth conditions and elicits few transcriptional changes. It is possible that expression of basal levels of HtrA under unstressed growth conditions could represent a mechanism to quickly respond to aberrant SrtA activity in the event of sudden environmental changes or fluctuations. This would parallel the role of HtrA in *S*. *pneumoniae*, where HtrA monitors protein secretion also during unstressed conditions [58,59]. While a limitation of the present study is that we have not identified the exact molecular mechanism of HtrA-mediated pilus clearance in *E*. *faecalis*, our results using a protease-defective HtrA suggests that clearance of aberrant membrane-anchored Ebp is carried out by the *E*. *faecalis* HtrA chaperone rather than by direct proteolytic degradation. One possibility is that the HtrA chaperone activity might facilitate interaction with other proteases or release of pili to the extracellular environment.

Many essential processes occur at the membrane, ranging from nutrient exchange and energy production, to interaction with the environment and protection from external insults. Thus, membrane homeostasis needs to be maintained to support proper functioning and cell viability. Aberrant accumulation of off-pathway pili triggers a membrane stress response in *E*. *coli* [15]. Here we show that accumulation of membrane-bound pili also elicits a global transcriptional response in *E*. *faecalis* that partially parallels the membrane stress response in *E*. *coli* [36]. Specifically, membrane overloading with pili in *E*. *faecalis* repressed non-essential lipoproteins (e.g. PTS and ABC-type transporters) and activated the aggregate-resolving ClpB chaperone as well as peptidoglycan-targeting enzymes (e.g. penicillin-binding proteins, peptidoglycan-active genes, murein hydrolase regulators). By contrast, *E*. *faecalis* did not upregulate genes involved in translation. It is possible that in Gram-positive bacteria such as *E*. *faecalis*, accumulation of membrane-bound pili elicits a milder membrane stress response than in Gram-negative bacteria. Here, it appears that the cell tries to return to membrane homeostasis by decreasing the non-essential protein load of the membrane, while redirecting cell resources into modifying the cell wall. It is possible that parallel upregulation of *liaXYZ* (but not *liaFSR*) prepares the cell to activate the LiaFSR membrane stress response if required. Notably, bacterial stress responses are known to hierarchically orchestrate adaptations depending on the degree of stress perceived, and the inability to activate CroRS was shown to constitutively induce *liaX* transcription [40]. Future studies are warranted to directly assess proteotoxic and membrane stress during defective pilus biogenesis. It is interesting to note that the initial proteotoxic stress in our study (i.e. inability to sort pili to the cell wall due to inactivation of *srtA*) likely transitioned into a membrane stress response due to the inability to remove membrane-bound pili by the HtrA quality control system. Indeed, deletion of *srtA* or *htrA* elicited only few transcriptional changes. While SrtA has 21 putative substrates in *E*. *faecalis* OG1RF [60], restoration of the morphology defect and partial restoration of dysregulated genes upon *ebpABC* deletion suggests than pili pose a significantly higher burden to the membrane than the rest of SrtA-dependent surface proteins. One possible explanation is that this is due to the high abundance of pilins that need to be secreted and assembled on the membrane before SrtA processing. Alternatively, it is possible that at least some of these surface proteins are not expressed under the tested conditions. Altogether, these results highlight the importance of SrtA and HtrA to avoid escalating a localized proteotoxic stress to a more generalized membrane stress.

An intriguing finding of this work is that the *ΔsrtA* and *ΔsrtAΔhtrA* strains produced more pili than the WT strain, and that this trait was dependent upon CroR activation. This is counterintuitive at first, since proteotoxic stress responses typically remove mistargeted proteins by upregulating chaperones and proteases, while downregulating synthesis of non-essential proteins (e.g. Ebp pilus) [13]. If CroRS evolved in part to counteract pilus-dependent membrane stress similar to *E*. *coli* Cpx, it would be expected to promote repression of *ebp* and activation of chaperones such as *htrA*. However, our data shows that CroR activation due to membrane-bound pili drives pilus expression, consequently perpetuating the source of endogenous stress. It is likely that CroRS is not directly involved in the response to proteotoxic stress. However, by inactivating the HtrA quality control system that normally clears proteotoxic stress, we uncovered a mild membrane stress response that is partially regulated by the CroRS TCS. In this scenario, CroR-dependent induction of pili and downstream biofilm formation could represent a protection strategy against membrane stressors. Overall, this leads us to propose the following model. During colonization and infection, pili are highly expressed but can be misfolded and aggregate as a result of harsh host conditions, impairing proper processing by sortase A [2,7,8,13]. Aberrant pili are continuously monitored and cleared (most likely indirectly) by HtrA, which is sufficient to resolve any proteotoxic stress that might arise. If the stress, however, is left unresolved and escalates into membrane stress, it triggers activation of CroRS, leading to hyper-piliation and activation of peptidoglycan remodeling pathways as protection mechanisms. It is currently unknown how CroS might sense cell membrane perturbations. In the closely-related *Streptococcus gordonii*, the TCS SGO_1180 was shown to monitor sortase A-dependent adhesin processing by directly sensing the remnant C-terminal LPXTG sorting motif that remains inserted in the membrane after efficient cell-wall anchoring [61]. Accumulation of the C-peptide that remains after sortase A processing inhibits activation of the TCS HK. BLASTP analysis suggests that SGO_1180 is not a CroS ortholog. However, it will be interesting to see if lack of pilus C-peptide is directly or indirectly linked to partial mobilization of the CroR regulon.

The crucial role that HtrA plays in preventing unproductive CroR activation due to membrane-bound pili is exemplified by the absence of a stress response in the hyper-piliated *ΔsrtA* strain. Aberrant activation of cell envelope stress responses are often associated with fitness costs for the cell [15,48], and CroR phosphorylation specifically has been shown to require tight control to avoid deleterious fitness defects [49], which argues in favor of not activating CroR unless required. This is especially true for *E*. *faecalis* OG1RF, a strain that harbors the CisRS TCS capable of phosphorylating CroR [49]. Therefore, it is possible that *E*. *faecalis* makes use of the HtrA quality control system to efficiently clear toxic pilus aggregates without eliciting a membrane stress response. In addition to pilus biogenesis, the Δ*htrA* strain has a minor defect in extracellular protease activity and our *in vivo* results suggest that HtrA promotes persistent wound colonization of *E*. *faecalis*. Previous work from our lab examined an *E*. *faecalis* OG1RF Δ*srtA*Δ*srtC* mutant strain in the wound colonization mouse model and observed no phenotype compared to the parental wild type strain, indicating that pili and other LPxTG cell wall-anchored surface proteins are dispensable for wound infection and persistence [25]. In line with this result, no significant differences were observed between the single Δ*htrA* mutant and the double Δ*ebpABC*Δ*htrA* strain 72h post-infection (**S6 Fig**), suggesting that HtrA promotes wound persistence independently of pili. Further studies are required to evaluate the significance of HtrA and CroRS to enterococcal colonization and infection beyond pilus biogenesis.

The CroRS TCS of *E*. *faecalis* has been predominantly studied in the context of cell wall-damaging antibiotics, where CroRS is a key determinant in *E*. *faecalis* resistance to cephalosporins (e.g. ceftriaxone). Recently, CroRS was shown to be important for cell wall homeostasis in the absence of antibiotics [40]. Our proposed model broadens the scope of the CroRS TCS from a cell wall stress responder to also a membrane stress responder. Basal cephalosporin resistance in *E*. *faecalis* is primarily mediated by the CroR-dependent Pbp4 and PbpA [39]. Considering that CroR is active, and *pbp4* and *pbpA* transcription is induced in the *ΔsrtAΔhtrA* mutant, it was puzzling that the strain was hyper-sensitive to cephalosporins nearing *croR*::*tn* values. However, the observation that inactivation of *croS* in this background did not revert the phenotype argues that the *ΔsrtAΔhtrA* strain cannot mount a full CroR response on the cell surface. It is possible that either absence of *srtA* or *htrA*, or that membrane perturbation in the double mutant strain, impairs proper localization or enzymatic function of membrane-bound penicillin binding proteins such as Pbp4 and PbpA, rendering the cell less tolerant even with an active CroR regulon and an induced *pbp4* and *pbpA* transcription. Interference with penicillin-binding proteins could also explain the slight daptomycin susceptibility of the *ΔsrtAΔhtrA* strain. Notably, work in *Staphylococcus aureus* showed that alterations in Pbp2 membrane localization determines susceptibility to β-lactams in constitutive daptomycin-resistant (i.e. LiaR active) cells, indicative of an overlap between CroRS and LiaFSR at the level of penicillin-binding proteins [62]. In this regard, the hyper-sensitivity of the triple OG1RF *croS*::*tnΔsrtAΔhtrA* is reminiscent of LiaFSR defective mutants [48]. Some *E*. *faecalis* strains, including OG1RF, possess the CisRS TCS which can phosphorylate CroR and to respond to cell wall stress [49]. Here we show that the CisS histidine kinase is also capable of signaling via the CroR response regulator during accumulation of aberrant membrane-bound pili. While the physiologic role of CisRS remains elusive, it appears to interact with other cell wall-related TCS such as CroRS [49] and VanRS [63] and to contribute to resistance to cell wall-active antibiotics. Future work is needed to elucidate why some *E*. *faecalis* strains maintain CisRS encoded in their chromosome and how this TCS integrates into the network of existing TCS.

**Fig 8:**
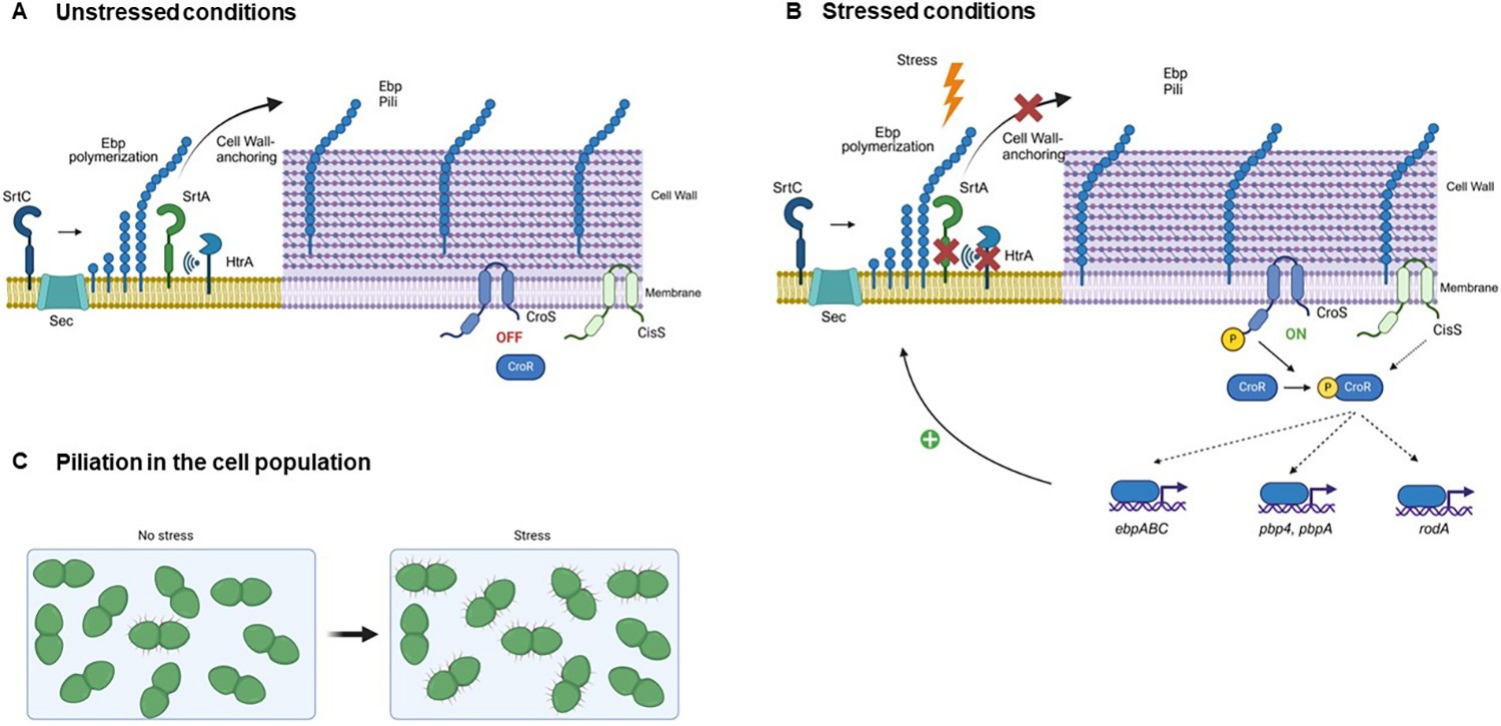
Model integrating the HtrA quality control system and the CroRS signalling pathway into pilus biogenesis. **(A)** During unstressed conditions, pilus monomers are exported through the Sec pathway, then the sortase C (SrtC) enzyme polymerizes EbpA (tip) and EbpC (shaft) subunits to full pilus length and finally links the structure to the EbpB (base) which remains transiently anchored to the cell membrane [28]. Then, the sortase A (SrtA) enzyme cleaves the CWS signal of EbpB and efficiently anchors the pilus to the cell wall [28,29]. Two models have been proposed for SrtA-dependent pili processing in *E*. *faecalis* [28,29]. The HtrA bifunctional chaperone/protease monitors pilus processing by SrtA and contributes to clearance of sporadic aberrant pili. **(B)** Certain environmental stresses (e.g. stomach pH, gut bile salts, immune oxidative burst) cause protein misfolding and aggregation, impairing successful processing by SrtA. If HtrA activation is insufficient to cope with the proteotoxic stress, aberrant proteins accumulate and perturb the cell membrane, leading to activation of the CroS histidine kinase. CroS in turn activates the CroR response regulator, which regulates transcription of pilus (*ebpABC*), penicillin-binding proteins (*pbp4*, *pbpA*), and peptidoglycan-active (*rodA*) genes among others. In some *E*. *faecalis* strains, the TCS CisRS can phosphorylate CroR [49]. Dotted arrows indicate confirmed indirect regulation (*pbpA*) [76] or unknown direct or indirect regulation by CroR. Induction of *ebpABC* genes leads to hyper-piliation, while repression of *rodA* partially leads to cell morphology alterations. (**C**) During non-stressed conditions, only a small percentage of cells expresses surface-exposed pili [2,4]. However, perturbation of the membrane due to accumulation of aberrant proteins during stress leads to piliation of a higher number of cells. Figure created with BioRender.com.

In this study we have uncovered an intricate link between the HtrA chaperone, the Ebp virulence factor, and the antibiotic response system CroRS (**Fig 8**). Our results confirm the role of HtrA as a pilus quality control system in *E*. *faecalis*, where it contributes to clearance of membrane-bound Ebp pili and avoids downstream membrane stress. We show that the CroRS TCS can be activated by endogenous (off-pathway pili) and possibly also by exogenous (daptomycin) membrane stress (as opposed to classic antibiotic-mediated cell wall damage), and that this leads to RodA-dependent cell morphology alterations and to pilus expression in an EbpR-independent manner. Further studies will be required to shed light on the role of HtrA during *E*. *faecalis* proteotoxic stress during colonization and/or infection and to elucidate its connection to CroRS.

## Materials and methods

### Ethics statement

We performed all approved procedures in accordance with the Institutional Animal Care and Use Committee (IACUC) in Nanyang Technological University, School of Biological Sciences (ARFSBS/NIEA0198Z) for murine wound infection model.

### Bacterial strains and general growth conditions

Bacterial strains and plasmids used in this study are listed in **S1 Table**. We inoculated *E*. *faecalis* from single colonies and grew cells statically at 37°C in Brain Heart Infusion (BHI) broth (Acumedia, USA) or agar (1.5%, Difco, US) for all assays unless otherwise stated. *E*. *coli* was grown in Luria Bertani (LB) broth (Difco, US) with shaking, or on agar plates at 37°C for DNA isolation and manipulation. To stimulate pilus expression in *E*. *faecalis*, strains were grown statically in Trypticase Soy Broth (Oxoid, UK) supplemented with 0.25% glucose (TSBG) and incubated at 37°C [2]. We used Müller Hinton (MH) broth and 1.5% agar to perform antibiotic susceptibility assays. All inoculations were cultured for 15 to 18 hours unless otherwise stated. When required, antibiotics were added at the following concentrations: for *E*. *coli*, Kanamycin (Km) 50 mg/L or Erythromycin (Em) 500 mg/L; for *E*. *faecalis* strains, Em 25 mg/L, Km 500 mg/L, Rifampicin (Rif) 25 mg/L, Chloramphenicol (Cm) 10 mg/L. All antibiotics were purchased from Sigma-Aldrich Corporation, USA.

### DNA manipulation and construction of deletion mutants and complement strains

We used the Wizard Genomic DNA Purification Kit (Promega, USA) to isolate bacterial genomic DNA from *E*. *faecalis* and the PureLink Quick Plasmid Miniprep Kit (Invitrogen, USA) to isolate plasmid DNA from *E*. *coli*. Primers used in the study are listed in **S2 Table**. Primer design was performed based on the annotated complete genome of *E*. *faecalis* OG1RF (NC_017316) [64]. Amplification of all gene products was performed using Phusion High-Fidelity DNA Polymerase (Thermo Scientific, USA); screening and validation of DNA sequences were performed using *Taq* DNA Polymerase (New England Biolabs, USA). T4 DNA ligase and restriction enzymes were purchased from New England Biolabs, USA. Ligation and restriction digestion were performed per respective manufacturer’s protocol. Plasmids were created by In-fusion cloning, where the In-fusion enzyme (Clontech, USA) fuses PCR-generated sequences containing a 15 bp overlap with linearized vectors on both ends of the insert via homologous recombination.

In-frame deletion of *htrA* (OG1RF_12305, new locus tag OG1RF_RS11805) was created according to a previously described method [65]. Briefly, we amplified regions approximately 450 bp upstream and downstream of the *htrA* gene from OG1RF using primer pair *htrA* sew-R/*ΔhtrA* del-F for the upstream region and *htrA* sew-F/*ΔhtrA* del-R for the downstream region. The amplified regions included the codons for the first 9 amino acids (coding for MHLLGGYFM) and the stop codon at the end of the *htrA* gene. These products were sewed together and amplified using *ΔhtrA* del-F/*ΔhtrA* del-R. We cloned the 900 bp PCR amplicon into pGCP213, a temperature sensitive Gram-positive shuttle vector [66] at the PstI restriction site to generate deletion construct p*delta*-*htrA*. We transformed the deletion construct into OG1RF wildtype, *ΔsrtA*, *croR*::*tn*, *croS*::*tn*, Δ*ebpABC* and OG1 Δ*cisS*Δ*croS* by electroporation and transformants were selected at 30°C on BHI agar containing Em. Single colonies were inoculated into individual tubes of BHI broth containing Em and passaged for two days at 30°C. Chromosomal integrant of this temperature-sensitive plasmid were selected by passaging the culture at 42°C in BHI, in the presence of Em. Selection for excision of the integrated plasmid by homologous recombination was accomplished by growing the bacteria at 30°C in the absence of Em. Loss of *htrA* locus in Em sensitive bacteria was verified by PCR using *ΔhtrA* del-F/*ΔhtrA* del-R and *htrA* cp-F/*ΔhtrA* del-R (screen). To create OG1RF *ΔsrtAΔhtrA*, *croS*::*tnΔhtrAΔsrtA*, *croR*::*tnΔhtrAΔsrtA* and OG1 *ΔcisSΔcroSΔsrtAΔhtrA* we transformed p*delta-srtA* [27] into *ΔhtrA*, *croS*::*tnΔhtrA*, *croR*::*tnΔhtrA*, and *ΔcisSΔcroSΔhtrA* respectively. Loss of *srtA* locus in Em sensitive bacteria was demonstrated by PCR using *ΔsrtA* del-F/*ΔsrtA* del-R. To obtain the quintuple *ΔsrtAΔebpABCΔhtrA* strain, the deletion construct p*delta*-*htrA* was transformed into the Δ*srtA*Δ*ebpABC* strain and screened for the absence of *htrA*. We constructed an *htrA* complementation vector by amplifying the *htrA* coding sequence plus 200 base pairs upstream of the *htrA* start codon to include its native promoter with *htrA* cp-F/*htrA* cp-R or *htrA* cp-F/*htrA* cp-R-HA. We digested the resultant 1.5 kb PCR product with EcoRI and BamHI and ligated into pGCP123, giving rise to p*htrA* or p*htrA-HA*. The p*ftsW/rodA* and p*croRcroS*-2xHA complementation vectors were constructed using the same strategy with primers described in S2 table. To construct an HtrA expression plasmid defective of protease activity, extracted p*htrA* was subjected to a single amino acid change on the conserved serine (S271) on *htrA* allele by site-directed mutagenesis (SDM) using S271A_F_SDM/S271A_R_SDM. To construct an EbpABC expression plasmid defective in pilus assembly, extracted p*ebpABCsrtC* was subjected to a single amino acid change on the conserved lysine (K186) of the *ebpC* allele using primer pairs EbpC_K186A_F/EbpC_K186A_R. Of note, the *srtC* gene was included in the complementation vector to avoid accumulation of unprocessed pilin monomers, likely due to saturation of the processing capacity of the native SrtC encoded on the chromosome as previously described [28]. All plasmids were sequenced for verification by standard Sanger sequencing (AIT biotech, Singapore). We verified the protein expression and stability of proteins in these expression plasmids by immunoblot using respective antibody immune sera (**S3 Table**).

### Growth kinetics at different temperatures

Overnight cultures were diluted 10-fold in BHI and grown for 1 h at 37°C. Cells were then normalized to OD_600_ of 0.003 in a final volume of 50 mL media. To test the growth kinetics under various temperatures, we incubated each set of cultures at 37°C, 42°C or 50°C. Cell culture was extracted hourly for measurement of OD_600_ and colony forming unit (CFU/mL) plating on BHI plates. The plates were incubated at 37°C aerobically for 16 h.

### Environmental stress tolerance assays

We prepared BHI plates containing NaCl (0.5 to 1.5 M) or H_2_O_2_ (1.0 to 2.5 mM; Sigma-Aldrich). For growth experiments involving pH, we adjusted the initial pH of BHI agar to pH 5.5, 6.0, and 7.0 with HCl before sterilization. 50 mM citrate-phosphate buffer of the desired pH was added to media after sterilization. Overnight cultures were spun down and washed with sterile 1× PBS. Cultures were resuspended in either the same or 1/10 of the original volume in 1× PBS and measured at OD_600_ using a spectrophotometer (UVmini-1240, Shimadzu, Japan). Cells were normalized to OD_600_ of 0.5 and 10-fold serial dilutions were made. 5 μL aliquot of each dilution was spotted on BHI agar plates containing different stressors and plates were incubated at 37°C aerobically for 48–72 h.

### Biofilm assays

Overnight cultures were normalized to OD_600_ of 0.5–0.6. Then, 8 μL of normalized culture were mixed with 200 μL of BHI or TSBG media in a 96-well plate (Nunc MicroWell 96-Well Microplates, Thermo Scientific, USA). To test the effects of temperature on biofilm formation, the plates were incubated at 30°C, 37°C or 42°C statically for 24 or 48 h. Following incubation, planktonic bacteria were discarded by tipping content into a waste container. The plates were washed twice with 1 x PBS to remove non-adherent bacteria before staining with 0.1% crystal violet for 15 mins at 4°C. The plate was then washed with PBS until negative control is clear. 200 μL of ethanol: acetone (4:1) was added per well and incubated on an orbital shaker for 30–60 mins with lid on to prevent evaporation. Absorbance was read at OD_595_ using the microplate reader (Infinite M200 Pro, Tecan, Switzerland). All biofilm assays were performed with three biological replicates, each with 12 technical replicates.

### Murine wound infection model

The murine wound infection model was carried out as described elsewhere [25]. Groups of four to five male C57BL/6 mice (7–8 weeks old, 22 to 25 g; InVivos, Singapore) were used. Briefly, bacterial strains were grown in 15 mL BHI media for 16–18 h at 37°C. Cells were pelleted, resuspended in 5 mL sterile 1× PBS and the OD_600_ was normalized to 2 × 10^8^ CFU/mL. For competitive infection, an equal volume of each strain was mixed prior to infection. Mice were euthanized at 8 or 72 hpi and one cm by one cm squared piece of skin surrounding the wound site was excised and collected in sterile 1× PBS. After homogenization, viable bacteria were enumerated by plating onto both BHI plates and antibiotic selection plates to ensure all recovered colony forming units corresponding to the inoculating strain. To measure the fitness of the strains in causing infection, we calculated the competitive index (CI) as described elsewhere [25].

### Bacterial cell fractionation

*E*. *faecalis* was grown to mid-log phase at the indicated temperatures and OD_600_ of 0.5, normalized to 0.6, and equivalent volumes subjected to centrifugation at 8,000 x *g* for 5 minutes. We washed the cell pellets once in PBS and digested for one hour with 10 mg/mL lysozyme from chicken egg white (Sigma-Aldrich, USA) in lysozyme buffer (10 mM Tris-HCl pH 8, 50 mM NaCl, 1 mM EDTA, 0.75 M Sucrose) yielding the whole cell lysate. For further fractionation, the lysate was further subjected to centrifugation at 20,000 x *g* for 5 minutes. The resulting supernatant containing material liberated from the cell wall digestion was designated the cell wall fraction and the pellet designated the protoplast fraction. All fractions were stored at -20°C until use.

### Immunoblotting

Whole cell lysates or bacterial fractions were boiled for at least 15 mins in NuPAGE LDS Sample Buffer (4×) with dithiothreitol (DTT) and SDS-PAGE was performed with NuPAGE Novex 3 to 8% Tris-acetate gels in NuPAGE Tris-acetate SDS running buffer (Life Technologies Corp., USA) to resolve proteins >150kDa. For smaller proteins, NuPAGE Novex 4–12% Bis-Tris gels in NuPAGE MOPS SDS running buffer were used. The iBlot Transfer Stacks were used to transfer proteins on the iBlot Gel Transfer Device (Life Technologies Corp. USA). We blocked the membrane with 3% P-Bovine Serum Albumin (P-BSA) for one hour at room temperature or at 4°C overnight and then incubated with the indicated anti-sera for two hours at room temperature or at 4°C overnight, with gentle shaking. Blots were washed and then incubated with Pierce horseradish peroxidase-conjugated secondary antibodies (Thermo Fisher Scientific, Inc., USA) and incubated with Super Signal West Femto or Pico chemiluminescent substrate (Thermo Fisher Scientific, Inc., USA). We processed the Green X-ray film (Carestream, USA) with a Kodak processor (Kodak X-OMAT processor 2000). The PageRuler Prestained Protein Ladder, 10 to 180 kDa (Thermo Scientific, USA) was used to monitor protein sizes. Polyclonal antisera were generated commercially (SABio, Singapore) by immunization of hosts (**S3 Table**) with purified recombinant proteins, except for rabbit monoclonal anti-Hemagglutinin purchased from Thermo Scientific, USA. SecA, EbpA, and EbpB were generated previously [27].

### Casein agar plate assay

To prepare casein agar plates, BHI agar and skim milk (Sigma-Aldrich, USA) were separately autoclaved and then combined in equal amounts to a final concentration of 2.5% skim milk. Antibiotics were supplemented into the agar when necessary. 20 mL of agar was then dispensed into each Petri dish before being allowed to solidify. To perform the assay, overnight cultures of bacterial strains were normalized to OD 1.0 in 1×PBS and 5 μL of normalized cultures were spotted onto casein agar plates before incubation at 37°C for 24 hours. Presence of secreted proteolytic activity was observed as a clear halo around the bacterial colony after a 24-hour incubation. Images of the agar plates were captured using the ProtoCOL3 Plus automated colony counter (Synbiosis, UK) and the annular radius was measured using ImageJ.

### Bacterial cell preparation for immunofluorescence staining

*E*. *faecalis* strains grown in TSBG to mid-log phase were washed and normalized as described above. The cells were fixed with fresh 3% paraformaldehyde at 4°C for 10 mins and smeared on poly-L-lysine pre-coated slides (Polysciences, Inc., USA). Cells were washed once with 1× PBS and incubated with 100 times dilution of BODIPY FL vancomycin (Van-FL) (Thermo Fisher Scientific, Inc., USA) at a final concentration of 5 ng/μL and incubated for one hour at room temperature in the dark. To visualize DNA, we added DAPI stain to the fixed cells at a final concentration of 2.5 ng/μL and incubated for 15 mins at room temperature in the dark. Cells were then blocked with filtered 2% P-BSA prior to adding 20 μL of respective primary antibody on to fixed cells and incubated overnight in 4°C, shaking. The primary antibody was paired with the respective Alexa Fluor labelled secondary antibody and incubated at room temperature for one hour. Finally, we mounted the slide with mounting media (Vectashield, USA) and coverslip for 30 mins before imaging or stored at 4°C in the dark prior to imaging with super-resolution structured illumination microscopy (SR-SIM) (Carl Zeiss, Germany) or inverted epi-fluorescence microscopy (Zeiss Axio Observer Z1, Germany). For quantification of piliation and mean fluorescence intensity, strains were imaged and analyzed in triplicate, choosing 6–7 representative fields in each run with on average 68–108 total cells per field. To visualize chain length, 5 μL of the culture was mixed with an equal volume of low melting agar (BioWorld, USA) on a glass slide before covering it with a coverslip. The slides were visualized using a phase contrast microscope (Zeiss Axio Observer Z1; Carl Zeiss GmbH) fitted with a 100× oil immersion objective with a numerical aperture 1.4 optovar 1.0 magnification changer 1.5×. Images were collected with AxioVision (Carl Zeiss Zen 8.0 and analyzed with ImageJ (http://rsb.info.nih.gov/ij/).

### Transmission electron microscopy

For ultrastructural analysis, we grew *E*. *faecalis* strains overnight and sub-cultured 1:10 into 20 mL TSBG media and grew cells to mid-log phase. We fixed the bacteria in 2% paraformaldehyde/ 2.5% glutaraldehyde in 100 mM phosphate buffer, pH 7.4 for one hour at room temperature, washed in phosphate buffer, and post-fixed in 1% osmium tetroxide (Polysciences Inc.) for one hour. Samples were then rinsed extensively in deionized water prior to bloc staining with 1% aqueous uranyl acetate (Ted Pella Inc., Redding, CA) for one hour. Following several rinses in deionized water, we dehydrated the samples in a graded series of ethanol and embedded in Eponate 12 resin (Ted Pella Inc.). Sections of 95 nm were cut with a Leica Ultracut UCT ultramicrotome (Leica Microsystems Inc., Bannockburn, IL), stained with uranyl acetate and lead citrate, and viewed on a JEOL 1200 EX transmission electron microscope (JEOL USA Inc., Peabody, MA) equipped with an AMT 8-megapixel digital camera and AMT Image Capture Engine V602 software (Advanced Microscopy Techniques, Woburn, MA).

### RNA purification for RNA sequencing

For RNA-Seq analysis of daptomycin-treated OG1RF WT, sample preparation was adapted from a previous study [67]. Briefly, bacterial overnights were sub-cultured in BHI + 50 mg/L CaCl_2_ and incubated to OD_600_ ~0.4 (log phase). Then, cells were exposed to sub-inhibitory concentrations of daptomycin (1μg/mL) for 15 min prior to RNA extraction. RNA extracted from untreated cells served as a control. For mRNA transcriptomic analyses of OG1RF WT, Δ*htrA*, Δ*srtA*, Δ*srtA*Δ*htrA*, *croR*::*tn*Δ*srtA*Δ*htrA* and Δ*srtAΔebpABC*Δ*htrA* we grew the bacteria overnight, statically in TSBG media at 37°C. The next morning, cultures were diluted 1:10 and grown to an optical density (600 nm) of 0.5. Total RNA was extracted using the UltraClean Microbial RNA Isolation Kit (MO BIO Laboratories Inc., Singapore). Extracted RNA samples were subjected to rigorous DNase treatment using TURBO DNA-free kit (Ambion, Singapore) and purified DNA-free RNA samples were subjected to ribosomal depletion with Ribo-Zero Magnetic Kits (Epicentre, Singapore), all according to manufacturer’s protocols. Quantification of RNA and DNA were performed using Qubit RNA Assay Kits and Qubit dsDNA HS Assay Kits (Invitrogen, Singapore), respectively. The integrity of RNA was analyzed by gel electrophoresis using Agilent RNA ScreenTape (Agilent Technologies, Singapore). RNA samples were prepared in triplicate from three independent biological samples. mRNA libraries for RNA sequencing were prepared using TruSeq Stranded mRNA Library Prep Kit (Illumina, USA), the quality of the library analyzed via Bioanalyzer (Agilent, USA), and sequencing performed using an Illumina Miseq V2 machine. RNA sequencing reads were mapped to the *E*. *faecalis* OG1RF reference genome (NCBI accession: NC_017316.1) using BWA (v0.5.9), or BWA-MEM2 (2.2.1) for *ΔsrtAΔebpABCΔhtrA*, *ΔsrtAΔhtrA* comparisons, with default parameters [68,69]. Sequencing reads (Accession numbers CP002621.1) mapping to predicted open reading frames (ORFs) were quantified using HTSeq [70]. Counts for ribosomal and transfer RNA sequences were filtered out of the data set and differential expression analyses were performed in R (version 2.15.1) using the Bioconductor package, edgeR [71]. Significantly differentially expressed genes were determined using a P-value and false discovery rate (FDR) cut-off of 0.05. We annotated differentially expressed genes using a combination of KEGG annotations, as well as manual annotation using operon and other functional data from the literature and *E*. *faecalis* OG1RF database available on BioCyc.

### Quantitative Real-time Polymerase Chain Reaction (qRT-PCR)

Quantitative reverse transcriptase PCR (qRT–PCR) was performed using a two-step method. 4900 ng of DNA-depleted RNA was first converted to complementary DNA (cDNA) using Superscript III First-Strand Synthesis System Kit (Invitrogen, USA). Following cDNA synthesis, 0.09 ng of cDNA per well was used in qRT-PCR with KAPA SYBR FAST qPCR Master Mix Kit (2×) (KAPA Biosystems, USA) on an Applied StepOnePlus Real-Time PCR System (Applied Biosystems, USA). No amplification was observed for no-template control in qPCR reaction (C_T_ value above 35). To compare the differences between the target genes, the ΔΔC_T_ method was used [72]. Prior to the ΔΔC_T_ analysis, qPCR data was validated by running a standard curve for each gene as described in Applied Biosystems User Bulletin No.2 (P/N 4303859) and elsewhere [72]. The housekeeping gene gyrase B (*gyrB*) was used as an endogenous control in this study [73,74]. Melting curve analyses were employed to verify the specific single-product amplification. Primers used in the study are listed in **S2 Table** and were generated using NCBI primer design software (Primer-BLAST) to amplify PCR products of size between 100–150 bp.

### Antibiotic susceptibility determinations

E-test (ETEST, BioMérieux, USA) MICs were determined with 1.5% MH agar using the incubation condition as described above. Briefly, bacteria from stationary phase cultures in MHB media were washed and normalized visually in 1× PBS to McFarland standard 1.0. E-test inoculum preparation, plating, strip application, and MIC determinations were carried out according to the manufacturer’s protocol [75].The antibiotics tested were vancomycin, daptomycin, ceftriaxone, and cefotaxime. Insufficient growth of bacteria on the agar plate to form a bacteria lawn for accurate MIC determination after 18 h incubation will be given an additional incubation time of not more than 24 h.

### Statistical analyses

Data from multiple experiments were pooled. Statistical significance for biofilm assay was determined using a two-tailed unpaired t-test. Statistical significance for *in vivo* animal experiments was determined using one-way ANOVA, Kruskal–Wallis test with Dunn’s post-test to correct for multiple comparisons. Statistical significance with the relative density of protein level was determined using Tukey’s multiple comparisons test. Statistical significance with the percentage of cells expressing pilin was determined using the Holm-Sidak method. Statistical significance with the number of division septa per cell unit was determined using the Holm-Sidak method. Statistical significance with the qRT-PCR was determined using Tukey’s multiple comparisons test. Unless otherwise stated, values represented means ± SEM derived from at least three independent experiments. * P ≤ 0.05; ** P ≤ 0.01; *** P ≤ 0.001; **** P ≤ 0.0001; P ≥ 0.05, differences not significant (ns). GraphPad Prism 7 software (GraphPad Software, La Jolla, CA) was used for statistical analyses.

## Supporting information

S1 Fig HtrA does not play a role in biofilm formation or growth in response to *in vitro* environmental stresses. **(A)** Incubation of *E*. *faecalis* WT and Δ*htrA* strains at 50°C (n = 3). Growth was monitored in BHI broth. CFU counts (CFU ml^-1^) are represented as dashed lines; OD_600_ readings are represented as solid lines. Standard deviation is indicated by bars. **(B)** Biofilm formation of WT and Δ*htrA* strains in either BHI or TSBG after 24h or 48h incubation at 30°C, 37°C, or 42°C. The values represent the mean values ± standard deviation obtained from two independent experiments, each with 12 technical replicates. **(C)** Summary of conditions tested to assay the stress tolerance of the Δ*htrA* mutant in response to variations in temperature, pH, osmolarity, and H_2_O_2_. Growth was assessed on conditioned BHI agar plates after 48h-72h incubation.
(TIF)

S2 Fig EbpA and EbpB accumulate in the protoplast fraction of the *ΔsrtAΔhtrA* strain and confirmation of a HtrA protease inactive variant. **(A)** Immunoblot was performed with α-EbpA or α-EbpB on protoplast fractions of WT, *ΔhtrA*, *ΔsrtA*, and *ΔsrtAΔhtrA* strains as well as in *ΔsrtAΔhtrA* strains carrying p*Empty* (vector control), p*srtA* or p*htrA*. The blot shows pilus HMWL. **(B)** Casein agar plate assay of bacterial strains after 24 hour incubation. Bacterial strains harbouring the indicated plasmids were assessed on agar plates supplemented with kanamycin. Photographs shown are representative of three independent experiments. Scale bar, 7 mm. (**C**) Annular radius of the clear zone of hydrolysis from the casein agar plate assay. Data from three independent experiments are shown and statistical analysis was performed using the 1-way ANOVA and Tukey’s comparison test. ** P ≤ 0.01; ***P ≤ 0.001; **** P ≤ 0.0001.
(TIF)

S3 Fig Hyperpiliation of the *ΔsrtAΔhtrA* strain occurs mainly on the population level. (A) Representative IF labelling of EbpC in *E*. *faecalis* WT and Δ*srtA*Δ*htrA* strains at mid-log phase. Cells were labeled with α-EbpC immune serum and Alexa Fluor 568 secondary antibody. Scale bar, 1 μm. (B) Quantification of percent EbpC^+^ cells in the population and mean EbpC fluorescence intensity of single EbpC^+^ cells. Strains were imaged and analyzed in triplicate, choosing 6–7 representative fields in each run with on average 68–108 total cells per field. EbpC fluorescence intensity is quantified in arbitrary units (AU) provided by the equipment. Results are represented as bar graphs with individual data points per field and standard error of mean. Combined data from three independent experiments are shown. Statistical analysis was performed by unpaired t test using GraphPad. ** P ≤ 0.01; P > 0.05, differences not significant (ns).
(TIF)

S4 Fig Pilus expression in the *croR*::Tn*ΔsrtAΔhtrA* strain. **(A)** Immunoblot was performed with α-EbpC on protoplast fractions of WT, *ΔhtrA*, *ΔsrtA*, *ΔsrtAΔhtrA*, *croR*::Tn and *croR*::Tn*ΔsrtAΔhtrA* strains. Top blot shows pilus HMWL and bottom blot shows loading control using α-SecA. Relative EbpC density differences were calculated with WT or *ΔsrtA* EbpC expression as the standard. **(B)** Statistical analysis of pilus-expressing cells of WT, *ΔhtrA*, *ΔsrtA*, *ΔsrtAΔhtrA*, and *croR*::Tn*ΔsrtAΔhtrA* strains as well as *croR*::Tn*ΔsrtAΔhtrA* + *pcroRS-HA* labeled with α-EbpC immune serum and Alexa Fluor 568 secondary antibody. Mean results are represented as bar graphs with standard error of mean. Combined data from three independent experiments are shown. * P ≤ 0.05; ***P ≤ 0.001; **** P ≤ 0.0001.
(TIF)

S5 Fig Absence of *srtA* and *htrA* result in aberrant cell morphology. **(A) The relative cell length of the bacterial population was determined by measuring the mid-length of the cells using PSICIC and ImageJ.** The data were plotted using Microsoft Excel. Cells that were not in-phase were excluded from the analysis. A total of at least 200 cells were sampled per strain. (B) The number of cell septation(s) per cell are represented as bars. **P < 0.01; ****P < 0.0001. The data was plotted using GraphPad Prism. **A total of at least 500 cells was sampled per strain. (C)** The cells were processed for TEM as described in the materials and methods section. Image representations of *ΔsrtAΔhtrA* cell structures. Scale bar represents 500 nm. **(D)** Immunoblot was performed with α-EbpC immune serum on protoplast fractions of WT, *ΔsrtAΔhtrA*, *ΔsrtAΔebpABCΔhtrA;* and *ΔsrtAΔebpABCΔhtrA* strains carrying p*Empty*, p*ebpABC* or p*ebpABC*^*K186A*^*srtC*. Top blot shows pilus high molecular weight ladders (HMWL) in *ΔsrtAΔhtrA* and *ΔsrtAΔebpABCΔhtrA* strains carrying p*ebpABCsrtC* as well as EbpC low molecular bands in *ΔsrtAΔebpABCΔhtrA* strains carrying p*ebpABC*^*K186A*^*srtC*, likely reflecting EbpAC or EbpBC doublets as described previously [27]. Bottom blot shows SecA immunoblot as a loading control.
(TIF)

S6 Fig HtrA supports *E*. *faecalis* persistence in wounds in a pili-independent manner. Wounds were infected with a 1:1 ratio of *E*. *faecalis* strains OG1X/OG1RF Δ*htrA* or OG1X/OG1RF Δ*ebpABC*Δ*htrA*, at 10^6^ CFU per inoculum, and harvested at 72 hpi. Recovered bacteria were enumerated on selective media for each strain. Each dot represents a mouse. Competitive index was calculated using the final CFU ratio of OG1X with OG1RF Δ*htrA* or Δ*ebpABC*Δ*htrA* (output) over the initial CFU ratio of OG1X with OG1RF Δ*htrA* or Δ*ebpABC*Δ*htrA* (input). Solid horizontal line indicates the median. N = 1, n = 5 mice. Statistical analysis was performed using the Mann-Whitney test.
(TIF)

S1 Table Bacterial strains and plasmids used in this study. (PDF)

S2 Table Primers used in this study. (PDF)

S3 Table Antibodies used in this study. (PDF)

S4 Table List of differentially expressed genes in Δ*htrA*, Δs*rtA*, and Δ*srtA*Δ*htrA* strains when compared to the WT strain. RNA-Seq of cells harvested at mid-log exponential phase incubated in TSB medium supplemented with glucose.
(XLSX)

S5 Table Core gene set of differentially expressed genes in the Δ*srtA*Δ*htrA* strain. List of differentially expressed genes in the Δ*srtA*Δ*htrA* strain when compared to WT, Δ*htrA* and Δs*rtA*. RNA-Seq of cells harvested at mid-log exponential phase incubated in TSB medium supplemented with glucose.
(XLSX)

S6 Table List of differentially expressed genes in the Δ*srtA*Δ*ebpABC*Δ*htrA* strain when compared to Δ*srtA*Δ*htrA*. RNA-Seq of cells harvested at mid-log exponential phase incubated in TSB medium supplemented with glucose.
(XLSX)

S7 Table Differential expression of the core gene set upon Ebp pili removal. RNA-Seq of cells harvested at mid-log exponential phase incubated in TSB medium supplemented with glucose.
(XLSX)

S8 Table List of differentially expressed genes in the *croR*::tnΔ*srtA*Δ*htrA* strain when compared to Δ*srtA*Δ*htrA*. RNA-Seq of cells harvested at mid-log exponential phase incubated in TSB medium supplemented with glucose.
(XLSX)

S9 Table List of differentially expressed genes in the *E*. *faecalis* OG1RF WT strain treated with 1μg/mL daptomycin. RNA-Seq of cells harvested at mid-log exponential phase incubated in BHI.
(XLSX)

S10 Table Raw data underlying graphs. (XLSX)
