# Supplementary figures and images for: The HtrA chaperone monitors sortase-assembled pilus biogenesis in Enterococcus faecalis
Source: PLoS Genet. 2024 Aug 5;20(8):e1011071. doi: 10.1371/journal.pgen.1011071 (PMC11326707; doi:10.1371/journal.pgen.1011071)

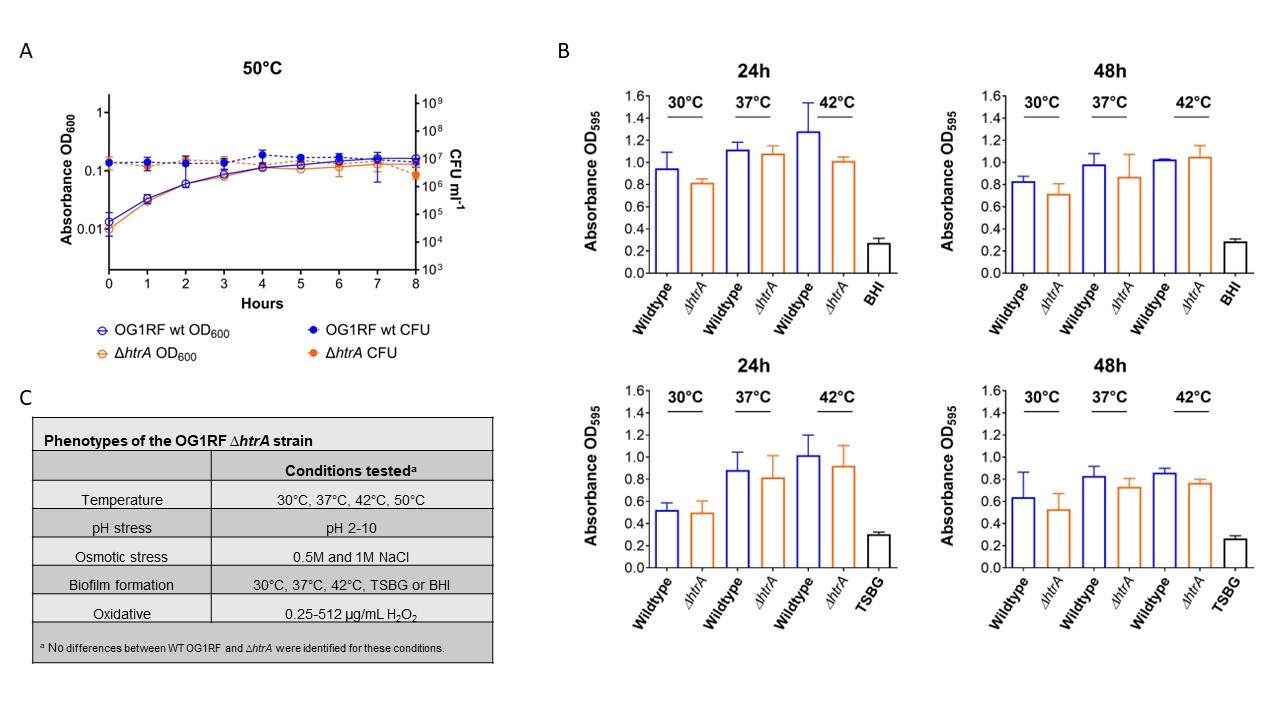

Supplement: S1 Fig — (A) Incubation of E. faecalis WT and ΔhtrA strains at 50°C (n = 3). Growth was monitored in BHI broth. CFU counts (CFU ml-1) are represented as dashed lines; OD600 readings are represented as solid lines. Standard deviation is indicated by bars. (B) Biofilm formation of WT and ΔhtrA strains in either BHI or TSBG after 24h or 48h incubation at 30°C, 37°C, or 42°C. The values represent the mean values ± standard deviation obtained from two independent experiments, each with 12 technical replicates. (C) Summary of conditions tested to assay the stress tolerance of the ΔhtrA mutant in response to variations in temperature, pH, osmolarity, and H2O2. Growth was assessed on conditioned BHI agar plates after 48h-72h incubation. (TIF) [file pgen.1011071.s001.tif]

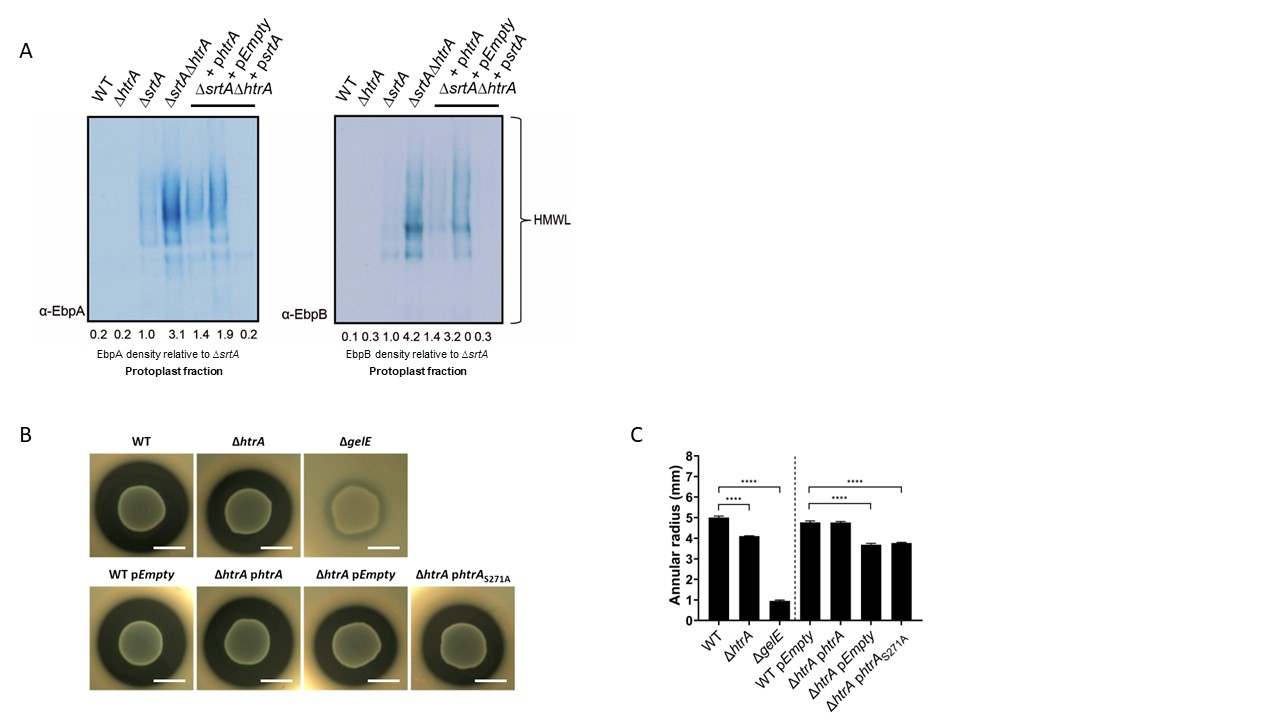

Supplement: S2 Fig — (A) Immunoblot was performed with α-EbpA or α-EbpB on protoplast fractions of WT, ΔhtrA, ΔsrtA, and ΔsrtAΔhtrA strains as well as in ΔsrtAΔhtrA strains carrying pEmpty (vector control), psrtA or phtrA. The blot shows pilus HMWL. (B) Casein agar plate assay of bacterial strains after 24 hour incubation. Bacterial strains harbouring the indicated plasmids were assessed on agar plates supplemented with kanamycin. Photographs shown are representative of three independent experiments. Scale bar, 7 mm. (C) Annular radius of the clear zone of hydrolysis from the casein agar plate assay. Data from three independent experiments are shown and statistical analysis was performed using the 1-way ANOVA and Tukey’s comparison test. ** P ≤ 0.01; ***P ≤ 0.001; **** P ≤ 0.0001. (TIF) [file pgen.1011071.s002.tif]

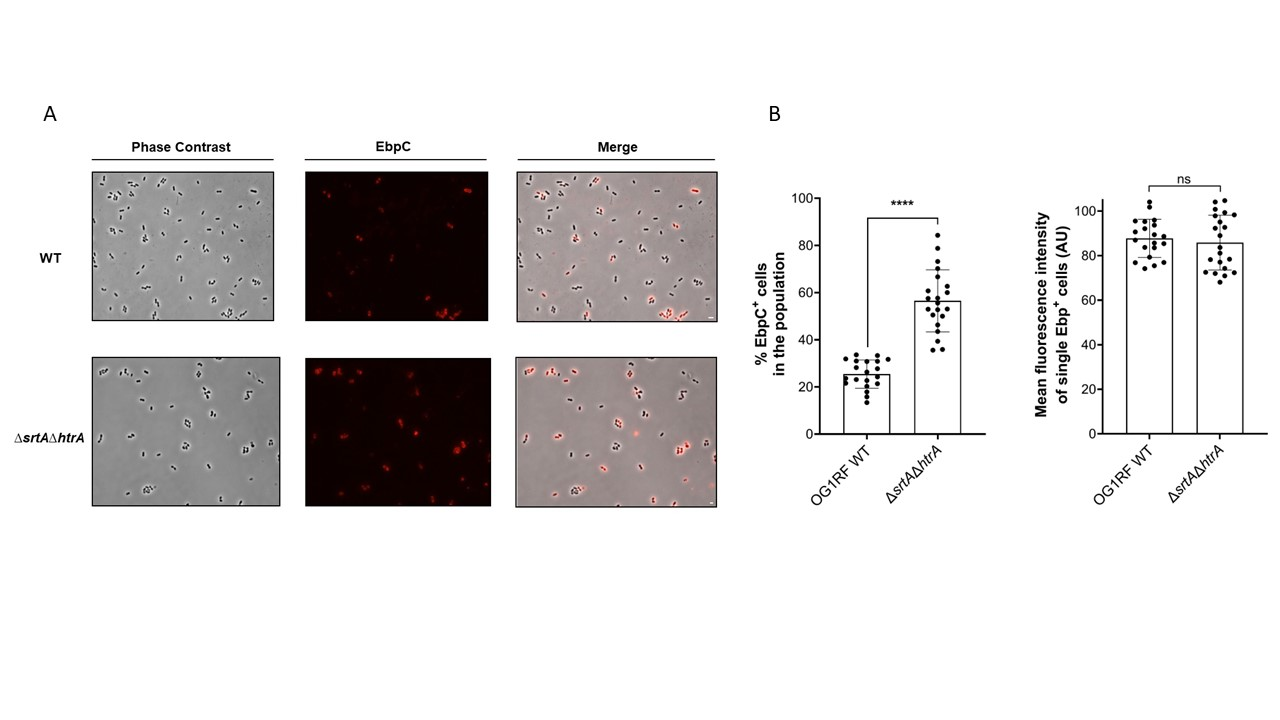

Supplement: S3 Fig — (A) Representative IF labelling of EbpC in E. faecalis WT and ΔsrtAΔhtrA strains at mid-log phase. Cells were labeled with α-EbpC immune serum and Alexa Fluor 568 secondary antibody. Scale bar, 1 μm. (B) Quantification of percent EbpC+ cells in the population and mean EbpC fluorescence intensity of single EbpC+ cells. Strains were imaged and analyzed in triplicate, choosing 6–7 representative fields in each run with on average 68–108 total cells per field. EbpC fluorescence intensity is quantified in arbitrary units (AU) provided by the equipment. Results are represented as bar graphs with individual data points per field and standard error of mean. Combined data from three independent experiments are shown. Statistical analysis was performed by unpaired t test using GraphPad. ** P ≤ 0.01; P > 0.05, differences not significant (ns). (TIF) [file pgen.1011071.s003.tif]

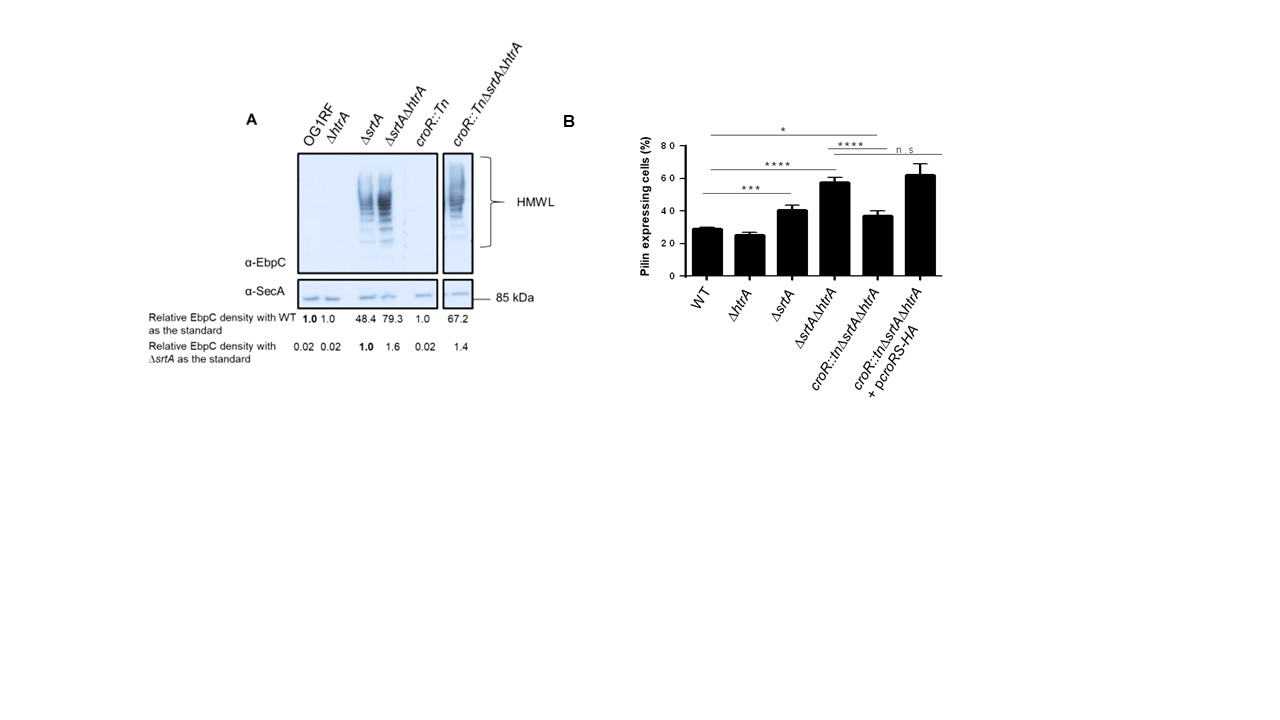

Supplement: S4 Fig — (A) Immunoblot was performed with α-EbpC on protoplast fractions of WT, ΔhtrA, ΔsrtA, ΔsrtAΔhtrA, croR::Tn and croR::TnΔsrtAΔhtrA strains. Top blot shows pilus HMWL and bottom blot shows loading control using α-SecA. Relative EbpC density differences were calculated with WT or ΔsrtA EbpC expression as the standard. (B) Statistical analysis of pilus-expressing cells of WT, ΔhtrA, ΔsrtA, ΔsrtAΔhtrA, and croR::TnΔsrtAΔhtrA strains as well as croR::TnΔsrtAΔhtrA + pcroRS-HA labeled with α-EbpC immune serum and Alexa Fluor 568 secondary antibody. Mean results are represented as bar graphs with standard error of mean. Combined data from three independent experiments are shown. * P ≤ 0.05; ***P ≤ 0.001; **** P ≤ 0.0001. (TIF) [file pgen.1011071.s004.tif]

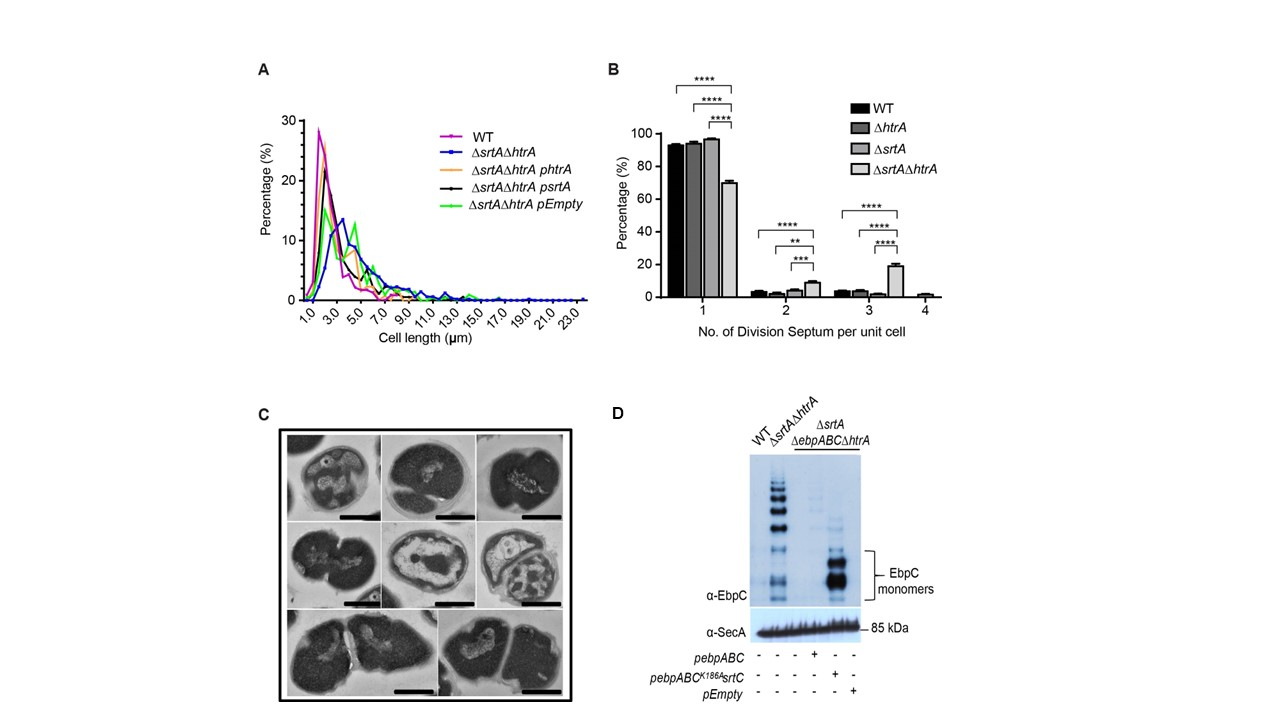

Supplement: S5 Fig — (A) The relative cell length of the bacterial population was determined by measuring the mid-length of the cells using PSICIC and ImageJ. The data were plotted using Microsoft Excel. Cells that were not in-phase were excluded from the analysis. A total of at least 200 cells were sampled per strain. (B) The number of cell septation(s) per cell are represented as bars. **P < 0.01; ****P < 0.0001. The data was plotted using GraphPad Prism. A total of at least 500 cells was sampled per strain. (C) The cells were processed for TEM as described in the materials and methods section. Image representations of ΔsrtAΔhtrA cell structures. Scale bar represents 500 nm. (D) Immunoblot was performed with α-EbpC immune serum on protoplast fractions of WT, ΔsrtAΔhtrA, ΔsrtAΔebpABCΔhtrA; and ΔsrtAΔebpABCΔhtrA strains carrying pEmpty, pebpABC or pebpABCK186AsrtC. Top blot shows pilus high molecular weight ladders (HMWL) in ΔsrtAΔhtrA and ΔsrtAΔebpABCΔhtrA strains carrying pebpABCsrtC as well as EbpC low molecular bands in ΔsrtAΔebpABCΔhtrA strains carrying pebpABCK186AsrtC, likely reflecting EbpAC or EbpBC doublets as described previously [27]. Bottom blot shows SecA immunoblot as a loading control. (TIF) [file pgen.1011071.s005.tif]

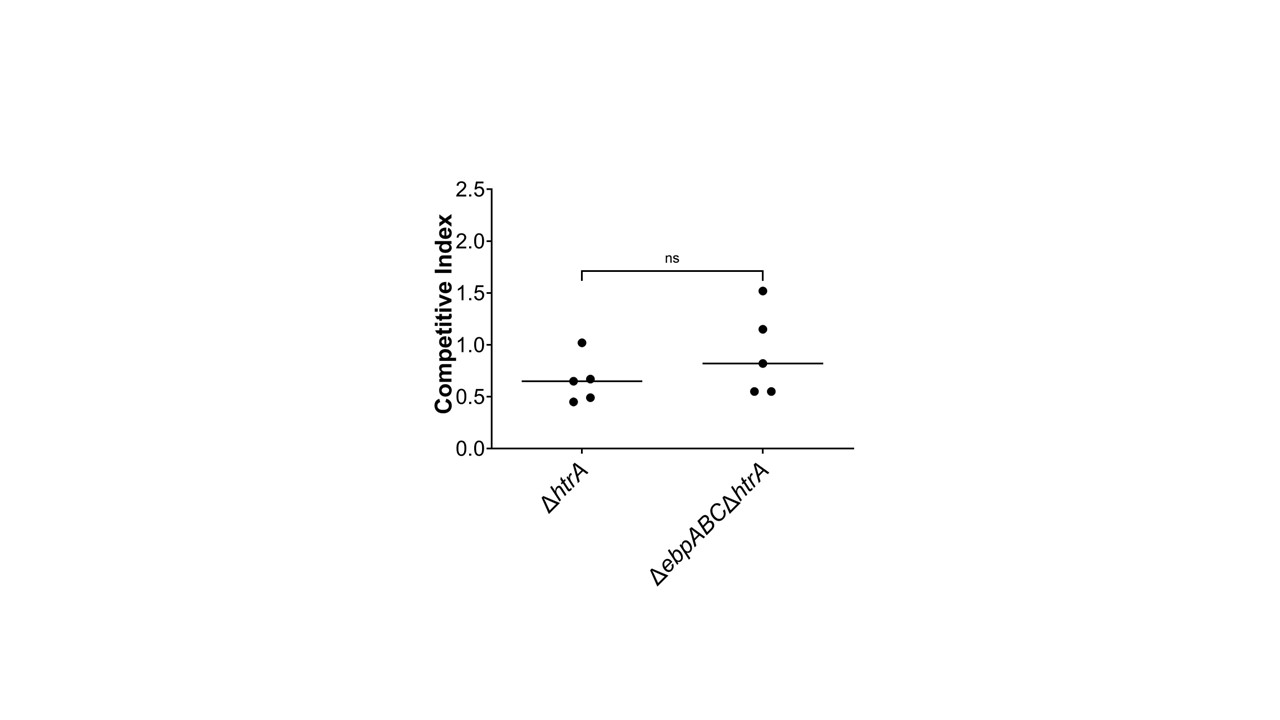

Supplement: S6 Fig — Wounds were infected with a 1:1 ratio of E. faecalis strains OG1X/OG1RF ΔhtrA or OG1X/OG1RF ΔebpABCΔhtrA, at 106 CFU per inoculum, and harvested at 72 hpi. Recovered bacteria were enumerated on selective media for each strain. Each dot represents a mouse. Competitive index was calculated using the final CFU ratio of OG1X with OG1RF ΔhtrA or ΔebpABCΔhtrA (output) over the initial CFU ratio of OG1X with OG1RF ΔhtrA or ΔebpABCΔhtrA (input). Solid horizontal line indicates the median. N = 1, n = 5 mice. Statistical analysis was performed using the Mann-Whitney test. (TIF) [file pgen.1011071.s006.tif]
