# Supplementary material for: The HtrA chaperone monitors sortase-assembled pilus biogenesis in Enterococcus faecalis
Source: PLoS Genet. 2024 Aug 5;20(8):e1011071. doi: 10.1371/journal.pgen.1011071 (PMC11326707; doi:10.1371/journal.pgen.1011071)
Supplement: S1 Table — (PDF) [file pgen.1011071.s007.pdf]

S1 Table. Bacterial Strains and Plasmids used in this study

Species and strain (antibiotic)[a]	Description	Reference
E. faecalis		
OG1RF	Fus^R and Rif^R derivative of wild-type OG1 oral isolate, reference sequenced lab strain	[1, 2]
OG1X	OG1 derivative, Str^R	[1]
Δ*gelE*	OG1RF *gelE* deletion	[3]
Δ*htrA*	OG1RF *htrA* deletion	This work
Δ*srtA*	OG1RF *srtA* deletion	[4]
Δ*srtA*Δ*htrA*	OG1RF *htrA srtA* deletion	This work
Δ*ebpABC*	OG1RF pilus negative strain	[5]
Δ*srtA*Δ*ebpABC*Δ*htrA*	OG1RF pilus negative, *htrA srtA* deletion	This work
Δ*ebpABC*Δ*htrA*	OG1RF pilus negative, *htrA* deletion	This work
croR::tn (Cm^R)	OG1RF with Tn insertion in *croR*	[6]
croS::tn (Cm^R)	OG1RF with Tn insertion in *croS*	[6]
*croR::tn*Δ*srtA*Δ*htrA* (Cm^R)	OG1RFΔ*srtA*Δ*htrA* with Tn insertion in *croR*	This work
*croS::tn*Δ*srtA*Δ*htrA* (Cm^R)	OG1RFΔ*srtA*Δ*htrA* with Tn insertion in *croS*	This work
Δ*cisS*Δ*croS*	OG1 *cisS croS* deletion	[7]
Δ*cisS*Δ*croS*Δ*srtA*Δ*htrA*	OG1 *cisS croS htrA srtA* deletion	This work
E. coli		
Stellar	*E. coli* host strain for routine cloning	Laboratory stock
DH5α	*E. coli* host strain for routine cloning	Laboratory stock
Plasmids		
pGCP123 (Kan)	Gram-positive expression vector (empty)	[8]
pGCP213 (Erm)	Temperature-sensitive plasmid for generation of deletions	[8]
p*htrA*	P_{htrA} *htrA* in pGCP123	This work
p*delta-htrA*	pGCP213 carrying *htrA* deletion	This work
p*htrA*$_{S271A}$	P_{htrA} *htrA*$_{S271A}$ in pGCP123	This work
p*delta-srtA*	pGCP213 carrying *srtA* deletion	[4]
p*srtA*	P_{rofA} *srtA* in pAL1	[4]
p*ebpABCsrtC*	P_{ebpA} *ebpABC*-P_{srtC} *srtC* in pGCP123	[5]
p*ebpABC*$_{K186A}$*srtC*	P_{ebpA}*ebpABC*$_{K186A}$-P_{srtC} *srtC* in pGCP123	This work
p*ftsW/rodA*	P_{ftsW}*ftsW/rodA* in pGCP123	This work

p*croR croS-2xHA*	P_{croR} *croR croS-2xHA* in pGCP123	This work

[a]Fus fusidic acid; Rif rifampicin; Kan kanamycin; Erm erythromycin; Cm chloramphenicol; Str Streptomycin

References

1. Dunny GM, Brown BL, Clewell DB. Induced cell aggregation and mating in Streptococcus faecalis: evidence for a bacterial sex pheromone. Proc Natl Acad Sci U S A. 1978;75(7):3479-83. doi: 10.1073/pnas.75.7.3479. PubMed PMID: 98769; PubMed Central PMCID: PMCPMC392801.
2. Bourgogne A, Garsin DA, Qin X, Singh KV, Sillanpaa J, Yerrapragada S, et al. Large scale variation in Enterococcus faecalis illustrated by the genome analysis of strain OG1RF. Genome Biol. 2008;9(7):R110. Epub 20080708. doi: 10.1186/gb-2008-9-7-r110. PubMed PMID: 18611278; PubMed Central PMCID: PMCPMC2530867.
3. Thomas VC, Hiromasa Y, Harms N, Thurlow L, Tomich J, Hancock LE. A fratricidal mechanism is responsible for eDNA release and contributes to biofilm development of Enterococcus faecalis. Mol Microbiol. 2009;72(4):1022-36. Epub 20090421. doi: 10.1111/j.1365-2958.2009.06703.x. PubMed PMID: 19400795; PubMed Central PMCID: PMCPMC2779696.
4. Kline KA, Kau AL, Chen SL, Lim A, Pinkner JS, Rosch J, et al. Mechanism for sortase localization and the role of sortase localization in efficient pilus assembly in Enterococcus faecalis. J Bacteriol. 2009;191(10):3237-47. Epub 20090313. doi: 10.1128/jb.01837-08. PubMed PMID: 19286802; PubMed Central PMCID: PMCPMC2687161.
5. Nielsen HV, Flores-Mireles AL, Kau AL, Kline KA, Pinkner JS, Neiers F, et al. Pilin and sortase residues critical for endocarditis- and biofilm-associated pilus biogenesis in Enterococcus faecalis. J Bacteriol. 2013;195(19):4484-95. doi: 10.1128/JB.00451-13. PubMed PMID: 23913319; PubMed Central PMCID: PMCPMC3807452.
6. Kristich CJ, Nguyen VT, Le T, Barnes AM, Grindle S, Dunny GM. Development and use of an efficient system for random mariner transposon mutagenesis to identify novel genetic determinants of biofilm formation in the core Enterococcus faecalis genome. Appl Environ Microbiol. 2008;74(11):3377-86. Epub 20080411. doi: 10.1128/aem.02665-07. PubMed PMID: 18408066; PubMed Central PMCID: PMCPMC2423031.
7. Kellogg SL, Kristich CJ. Functional Dissection of the CroRS Two-Component System Required for Resistance to Cell Wall Stressors in Enterococcus faecalis. J Bacteriol. 2016;198(8):1326-36. Epub 20160331. doi: 10.1128/jb.00995-15. PubMed PMID: 26883822; PubMed Central PMCID: PMCPMC4859583.
8. Nielsen HV, Guiton PS, Kline KA, Port GC, Pinkner JS, Neiers F, et al. The metal ion-dependent adhesion site motif of the Enterococcus faecalis EbpA pilin mediates pilus function in catheter-associated urinary tract infection. mBio. 2012;3(4):e00177-12. Epub 20120724. doi: 10.1128/mBio.00177-12. PubMed PMID: 22829678; PubMed Central PMCID: PMCPMC3419518.
