# Supplementary material for: The HtrA chaperone monitors sortase-assembled pilus biogenesis in Enterococcus faecalis
Source: PLoS Genet. 2024 Aug 5;20(8):e1011071. doi: 10.1371/journal.pgen.1011071 (PMC11326707; doi:10.1371/journal.pgen.1011071)
Supplement: S2 Table — (PDF) [file pgen.1011071.s008.pdf]

S2 Table. Primers used in this study

Primers[a]	Sequence (5'→3')[b]	Note[c]
Construction of complementation plasmids		
htrA cp-F	GCTTGATACGAATTCATTCGATAGACTAAAGGAGTAG	EcoRI
htrA cp-R	TAGAACTAGTGGATCCTTAGAATTGCCTTTTGATGC	BamHI
croR cp-F	GCTTGATATCGAATTCGATTGGTATAGGTATTTTCGTTCGTTTTTTACCC	EcoRI
croS cp-R-HA	TTAAGCGTAATCTGGAACATCGTATGGGTAAGCGTAATCTGGAACATCGTATGGGTA ACTCTCTGATTTCTTGTTG	
ftsW_F	TTGATATCGAATTCCTGCAGCAGAAATGTGAGCAACACACAAAAATTATATAAATGTC T	PstI
rodA_R	TGGCGGCCGCTCTAGATCAAGCGTAATCTGGAACATCGT ATGGGTAA	XbaI
Construction of deletion mutants		
htrA sew-F	TTTATGCAACGAAAAGATGTTCGCAGCAATCAATAAAAGAAA	
htrA sew-R	TTTCTTTTATTGATTGCTGCGAACATCTTTTCGTTGCATAAA	
Δ*htrA* del-F	AAACTGCAGATCTTGGCATAGCTATTTAAACG	PstI
Δ*htrA* del-R	TCCAATGCATTGGCTGCAGAATACAGCGATTAAAGATGCG	PstI
Δ*htrA* del-R (screen)	AACTAGTGGATCCCTTTTACTAAGCATAATCTGGAACATCATATGGATATTGATTGCT GCGATTATTT	
Δ*srtA* del-F	CGATTGACGCTTTCTTCTCC	
Δ*srtA* del-R	GAAACAGCAAGAACGCCAAG	
Site-directed mutagenesis[d]		

ebpC$_{K186A}$-F	GTTGTTCATATTTATCCT**GC**AAATGTAGTAGCCAATGATG
ebpC$_{K186A}$-R	CTACATTT**GC**AGGATAAATATGAACAACCGCTAATTCTTC
htrA$_{S271A}$-F	GCCATTCAAACCGATGCTGCCATCAATCCAGGAAACGCTG
htrA$_{S271A}$-R	GACTTGTCCTTCAATATTGATTAGTGGACCACCAGCGTTT
Screening primers	
croR sc-F	ATTTCGTTCGAAGGAACTACA
croR sc-R	TATAGGGAGACCGGCCTCGAG
ebpC sc-F	AGCGGGAAAGAAATGAGCGA
ebpC sc-R	AACGCCACCACCATATTCGT
T7 Universal Promoter	TAATACGACTCACTATAGGG
T3 Universal Promoter	CAATTAACCCTCACTAAAGG
M13 (-20) F primer	GTAAAACGACGGCCAGTG
M13 (-40) R primer	CAGGAAACAGCTATGAC
RT-qPCR	
ebpA-F	GGATGGTCGCTTTTACGGGA
ebpA-R	GCCATTGCCTCACCTATCGT
ebpB-F	GCTACTCGCTCTTTTCGGGT
ebpB-R	CTTCCCCTGTGTTTTGCTGC
ebpC-F	CGGTCATACCGACGACCAAA
ebpC-R	TGTCACATCGCCATCGACTT

gyrB_F	CAAGCCAAAACAGGTCGCC
gyrB_R	ACCAACACCGTGCAAGCC
croR_F	ATTTTTGAGCGTGTGTGGCAA
croR_R	TCTCCACCAGTTGCTTCTTCAA
croS_F	AGCTGTTCTCTATTGGCGCT
croS_R	CAAACGGAATGCGGTGATTGT

[a]Primers were designed according to the genomic sequence of E. faecalis OG1RF (GenBank accession number CP002621.1). F, forward primer; R, reverse primer. [b]Complementary sequences to vector are in red. [c]Restriction sites underlined. [d]Site-directed mutagenesis residues are in **bold**.
