# Supplementary material for: The HtrA chaperone monitors sortase-assembled pilus biogenesis in Enterococcus faecalis
Source: PLoS Genet. 2024 Aug 5;20(8):e1011071. doi: 10.1371/journal.pgen.1011071 (PMC11326707; doi:10.1371/journal.pgen.1011071)
Supplement: S3 Table — (PDF) [file pgen.1011071.s009.pdf]

S3 Table. Antibodies used in this study

Antigen	*Size (kDa)*	*Primary ab conc; host*	*Secondary antibody conc; host*
EbpA	122.7	1:3000; Rabbit	1:5000; Goat anti-rabbit HRP-conjugate
EbpB	53.4	1:3000; Rabbit	1:5000; Goat anti-rabbit HRP-conjugate
EbpC	68.2	1:3000; Guinea pig	1:5000; Goat anti-guinea pig HRP conjugate
			1:500; Alexa Fluor 568 labeled goat anti-guinea pig
HA		1:3000; Rabbit	1:5000; Goat anti-rabbit HRP-conjugate
			1:500; Alexa Fluor 488 labeled goat anti-rabbit
HtrA	45.7	1:1000; Rat	1: 5000; Goat anti-rat HRP-conjugate
SecA	97.0	1: 3000; Rabbit	1: 5000 Goat anti-rabbit HRP-conjugate
SrtA	27.1	1: 250; Mouse	1:1250; Goat anti-mouse HRP-conjugate
